# Efficacy and safety of antibiotics targeting Gram-negative bacteria in nosocomial pneumonia: a systematic review and Bayesian network meta-analysis

**DOI:** 10.1186/s13613-024-01291-5

**Published:** 2024-04-25

**Authors:** David Luque Paz, Dara Chean, Pierre Tattevin, Damien Luque Paz, Betsega Assefa Bayeh, Achille Kouatchet, Delphine Douillet, Jérémie Riou

**Affiliations:** 1 Infectious Diseases and Intensive Care Unit, Pontchaillou Hospital, University Hospital of Rennes, 2, rue Henri Le Guilloux, 35033 Rennes Cedex 9, France; 2 Inserm U1230, Université de Rennes, Rennes, France; 3 Intensive Care Unit, University Hospital of Angers, Angers, France; 4 Laboratory of Hematology, Angers University Hospital, Angers, France; 5 INSERM, CRCINA, University of Angers, Angers, France; 6 Department of Pneumology and Respiratory Functional Exploration, University Hospital of Tours, Tours, France; 7 Emergency Department, Angers University Hospital, Angers, France; 8 University of Angers, UMR MitoVasc CNRS 6015 - INSERM 1083, Angers, France; 9 FCRIN, INNOVTE, Saint Etienne, France; 10 University of Angers, Inserm, CNRS, MINT, SFR ICAT, 49000 Angers, France; 11 Methodology and Biostatistics Department, Delegation to Clinical Research and Innovation, Angers University Hospital, Angers, France

**Keywords:** Nosocomial pneumonia, Ventilator-associated pneumonia, Gram-negative bacilli, Antibiotics, Network meta-analysis

## Abstract

**Background:**

Multiple randomized controlled studies have compared numerous antibiotic regimens, including new, recently commercialized antibiotics in the treatment of nosocomial pneumonia (NP). The objective of this Bayesian network meta-analysis (NMA) was to compare the efficacy and the safety of different antibiotic treatments for NP.

**Methods:**

We conducted a systematic search of PubMed, Medline, Web of Science, EMBASE and the Cochrane Library databases from 2000 through 2021. The study selection included studies comparing antibiotics targeting Gram-negative bacilli in the setting of NP. The primary endpoint was 28 day mortality. Secondary outcomes were clinical cure, microbiological cure and adverse events.

**Results:**

Sixteen studies encompassing 4993 patients were included in this analysis comparing 13 antibiotic regimens. The level of evidence for mortality comparisons ranged from very low to moderate. No significant difference in 28 day mortality was found among all beta-lactam regimens. Only the combination of meropenem plus aerosolized colistin was associated with a significant decrease of mortality compared to using intravenous colistin alone (OR = 0.43; 95% credible interval [0.17–0.94]), based on the results of the smallest trial included. The clinical failure rate of ceftazidime was higher than meropenem with (OR = 1.97; 95% CrI [1.19–3.45]) or without aerosolized colistin (OR = 1.40; 95% CrI [1.00–2.01]), imipemen/cilastatin/relebactam (OR = 1.74; 95% CrI [1.03–2.90]) and ceftazidime/avibactam (OR = 1.48; 95% CrI [1.02–2.20]). For microbiological cure, no substantial difference between regimens was found, but ceftolozane/tazobactam had the highest probability of being superior to comparators. In safety analyses, there was no significant difference between treatments for the occurrence of adverse events, but acute kidney failure was more common in patients receiving intravenous colistin.

**Conclusions:**

This network meta-analysis suggests that most antibiotic regimens, including new combinations and cefiderocol, have similar efficacy and safety in treating susceptible Gram-negative bacilli in NP. Further studies are necessary for NP caused by multidrug-resistant bacteria.

*Registration* PROSPERO CRD42021226603

**Supplementary Information:**

The online version contains supplementary material available at 10.1186/s13613-024-01291-5.

## Introduction

Over the two last decades, bacterial multidrug resistance (MDR) has emerged and spread widely all around the world. The burden of this issue was estimated to be around five million deaths associated with bacterial resistance in 2019 [1]. The World Health Organization emphasized that it represents one of the biggest threats to global health by putting the achievements of modern medicine at risk [2]. Among emerging MDR bacteria, Gram-negative bacilli (GNB) are at forefront of concerns [3]. Consequently, rates of infections due to third-generation cephalosporin-resistant *Enterobacterales* (3GCRE) have dramatically increased in most countries [4, 5].

New antibiotics have recently been developed to offer alternatives in the treatment of infections due to MDR or extensively drug resistant (XDR) GNB. However, the emergence of metallo-beta-lactamase and class D beta-lactamase producing bacteria has made antimicrobial treatment challenging despite the development of these new antibiotics.

Nosocomial pneumonia represents the second most frequent healthcare-associated infection [6], of which GNB are leading pathogens [7]. Nosocomial pneumonia (NP) includes hospitalized-acquired pneumonia (HAP) and ventilator-acquired pneumonia (VAP) which carry high attributable costs as well as high morbidity and mortality [8].

Four new antibiotics including three combinations of beta-lactam/beta-lactamase inhibitor (BLBLI)—namely ceftolozane/tazobactam, ceftazidime/avibactam, imipemen/cilastatin/relebactam—and cefiderocol have been assessed in separate randomized controlled trials (RCTs) and then commercialized in the treatment of nosocomial pneumonia [9–12]. Most of these RCTs demonstrated a non-inferiority *versus* comparator (meropenem or piperacillin/tazobactam), but no study compared theses different molecules face-to-face. In this Bayesian network meta-analysis (NMA), we aimed to assess the efficacy and safety of antibiotics targeting GNB used in the treatment of nosocomial pneumonia and compare them, in order to determine the best available treatment.

## Material and methods

This study was a systematic review with Bayesian NMA performed in accordance with the Preferred Reporting Items for Systematic Reviews and Meta-Analyses (PRISMA) recommendations statements [13]. The protocol was registered in the PROSPERO database prior to study initiation (CRD42021226603).

### Search strategy

Two authors (DLP, DC) conducted a systematic search of PubMed, Medline, Web of Science, EMBASE and the Cochrane Library databases from January 1st, 2000, to December 31st 2020 for studies comparing antibiotics in the treatment of NP, including VAP, with available data on outcomes in each group of treatment. We initially used a broad search strategy by using MeSH search terms detailed in the Additional file 6.

Among records identified, we restricted the search for articles written in English dealing with human adults. We excluded studies which were performed on animals or specific populations (pregnant women, children). We also excluded studies focusing on community-acquired pneumonia or with mixed infection types. In the remaining articles, we sought studies which reported mortality data for each group of antibiotic treatment. Particular attention was paid to the risk of duplicate reports, and whenever identified, duplicate studies were excluded.

### Eligibility criteria and study selection

First, we included RCTs, clinical trials, observational comparative studies that compared outcomes in patients who had received different antibiotics targeting GNB in confirmed nosocomial pneumonia. Inclusion and exclusion criteria are fully detailed in the Additional file 6. These studies had to provide 28 day mortality rates (or if not available, in-hospital or crude mortality rates with follow-up exceeding 14 day) for each treatment group. We did not include preprints or non-peer-reviewed works.

The articles were first screened by two authors (DLP, DC) independently based on title and abstract. Selected articles were assessed by full-text reviewing and studies fulfilling the predetermined inclusion criteria were included. In addition, reference lists of relevant articles were screened using the snowballing method. All disagreement over study inclusion led to discussion in order to find a consensus or, if necessary, were solved by the adjudication of a third author (DD). We excluded articles reporting subgroup analyses data that overlap with other studies. Participants, study design and comparisons of all included studies are described in Table 1.

**Table 1:** Baseline characteristics in trials retained for the final analysis

Author	Antibiotic regimen	Funding	Nb of patients*, N	Male gender, N	Age, mean ± SD	VAP, N	Bacteraemia	Apache II score, mean ± SD	Renal failure (Cl < 60 ml/min)	Documented pathogens (GNB)	*K. pneumoniae*	*E. coli*	*P. aeruginosa*	*A. baumanii*	No documentation
Titov et al	Imipenem/Cilastatin/Relebactam	Funded	264	178	60.5 ± 16.9	122	15	14.6 ± 6.2	71	215	58	30	34	32	49
	Piperacillin/Tazobactam	Funded	267	189	58.8 ± 18.4	136	16	14.8 ± 6.7	60	218	53	37	48	36	49
Zanetti et al	Cefepime	Funded	108	72	55 ± 18	66	*NA*	15.6 ± 6.6	*NA*	77	18	7	23	12	31
	Imipenem/Cilastatin	Funded	101	67	53 ± 18	66	*NA*	14.8 6.3	*NA*	71	16	2	29	8	31
Wunderink et al	Cefiderocol	Funded	145	99	64.6 ± 14.6	58	*NA*	16 ± 6.1	47	113	48	19	24	23	20
	Meropenem	Funded	147	101	65.4 ± 15.1	63	*NA*	16.4 ± 6.9	51	105	44	22	24	24	19
Torres et al	Ceftazidime/Avibactam	Funded	356	268	62.1 ± 16.6	118	19	14.5 ± 4	18	173	37	11	42	*NA*	183
	Meropenem	Funded	370	274	61.9 ± 17.4	128	15	14.9 ± 4	18	188	49	18	35	*NA*	182
Schmitt et al	Piperacillin/Tazobactam	Independent	110	77	68.4 ± 13.7	31	*NA*	13.5 ± 4.2	*NA*	*NA*	*NA*	*NA*	*NA*	*NA*	*NA*
	Imipenem/Cilastatin	Independent	111	64	65.7 ± 13.8	21	*NA*	13.3 ± 4.3	*NA*	*NA*	*NA*	*NA*	*NA*	*NA*	*NA*
Chastre et al	Doripenem	Independent	126	102	50.7 ± 19.6	126	13	*NA*	13	92	15	12	20	*NA*	34
	Imipenem/Cilastatin	Independent	122	91	50.3 ± 19	122	11	*NA*	8	82	10	10	14	*NA*	30
Kollef et al	Doripenem	Funded	115	72	57.5 ± 16.5	115	6	*NA*	13	65	19	6	17	15	50
	Imipenem/Cilastatin	Funded	112	75	54.6 ± 18.5	112	5	*NA*	8	62	20	14	10	10	50
Kollef et al	Ceftolozane/Tazobactam	Funded	362	262	60.5 ± 16.7	362	25	17.5 ± 5.2	52	157	86	51	63	24	98
	Meropenem	Funded	364	255	59.5 ± 17.2	364	40	17.4 ± 5.7	48	137	91	42	65	14	117
Cisneros et al	IV Colistin	Independent	120	92	63 (IQR: 49–71)	120	21	19 (IQR: 14–24)		89	14	12	19	16	19
	Meropenem	Independent	112	81	60.5 (IQR: 50–70)	112	17	17 (13–22)		85	15	8	15	18	19
Réa-Neto et al	Doripenem	Funded	134	98	57.5 ± 19.2	105	8	*NA*	21	92	20	14	22	14	*NA*
	Piperacillin/Tazobactam	Funded	119	74	59.3 ± 18.9	93	17	*NA*	9	98	28	11	32	9	*NA*
Yamamato et al	Piperacillin/Tazobactam	Funded	34	18	77.6	0	*NA*	*NA*	*NA*	19	6	2	2	*NA*	15
	Meropenem	Funded	33	18	79.1	0	*NA*	*NA*	*NA*	18	1	0	4	*NA*	15
Lerma et al	Meropenem	Funded	69	47	61.5 ± 13.7	69	*NA*	16.5 ± 5.7	*NA*	44	5	6	14	3	*NA*
	Ceftazidime	Funded	71	56	62.3 ± 15.7	71	*NA*	16.6 ± 6	*NA*	34	2	3	13	2	*NA*
Torres et al	Ciprofloxacin	Independent	41	31	64 ± 14	41	*NA*	13.8 ± 7.5	2	41	1	1	14	6	*NA*
	Imipenem/Cilastatin	Independent	34	25	61 ± 17	34	*NA*	13.9 ± 8.6	0	34	1	1	12	3	*NA*
Abdelsalam et al	Colistin (IV)	Independent	30	16	56.2 ± 17.9	30	*NA*	18.1 ± 4	2	30	30	0	0	0	0
	Meropenem + Colistin (AS)	Independent	30	12	55.9 ± 15.6	30	*NA*	18.9 ± 5.5	4	30	30	0	0	0	0
Alvarez-Lerma et al	Piperacillin/Tazobactam	Funded	88	64	57.1 ± 17	75	*NA*	16.5 ± 6.6	4	64	1	5	14	2	*NA*
	Ceftazidime	Funded	36	26	60.5 ± 20	31	*NA*	16.9 ± 6.5	2	29	1	2	7	0	*NA*
Joshi et al	Piperacillin/Tazobactam	Funded	222	173	53.2 ± 19.1	157	*NA*	13.9	*NA*	155	14	5	18	9	*NA*
	Imipenem/Cilastatin	Funded	215	138	52.7 ± 20.9	143	*NA*	13	*NA*	121	12	10	17	8	*NA*

### Assessment of risk of bias

Risk of bias (RoB) for studies included in the NMA was independently assessed by two authors (DLP, DC), and a third author (DD) was solicited to solve disagreements.

### Outcomes definition

The primary outcome was all causes 28 day mortality. Clinical cure, microbiological cure and the occurrence of adverse effects were assessed as secondary outcomes. Clinical cure was defined as resolution or substantial improvement of baseline symptoms/signs and the absence of additional antibiotic treatment at the end of treatment or test-of-cure visit. Microbiological cure was achieved when baseline Gram-negative pathogen(s) were eradicated or presumed to be eradicated on clinical culture specimen at the end of treatment of test-of-cure visit.

### Statistical analysis

In this study, a Bayesian network meta-analysis with an unconstrained random effect model was performed. Gibbs sampler in Bayesian hierarchical model with binomial prior distribution was used to assess the consistency of estimates. Odds Ratio (ORs) with their 95% credible interval (95% CrI) was used to summarize to treatment effect. Convergence of relative treatment effects, baseline effect, and heterogeneity parameter was tested using the Brooks-Gelman-Rubin statistics. A node-splitting model was performed for all loops of the network to detect the inconsistency between direct and indirect comparisons [14].

A probabilistic analysis was also realized and summarized using the surface under the cumulative ranking curve (SUCRA), and an overall ranking based on the probability that a treatment was the most effective for the outcome of interest. A subgroup analysis was performed according to risk of bias.

Furthermore, network meta-regressions were realized to assess different treatment effect: age, kidney failure, adjunctive use of amikacin, non-fermenting GNB, severity at randomization and study design.

All statistical analysis were performed using R software version 4.0.3 (R Foundation for Statistical Computing). These analyses required the use of the following R packages: “bnma”, “rjags”, and “ggplot2”.

## Results

### Characteristics of included trials

**Fig. 1:**
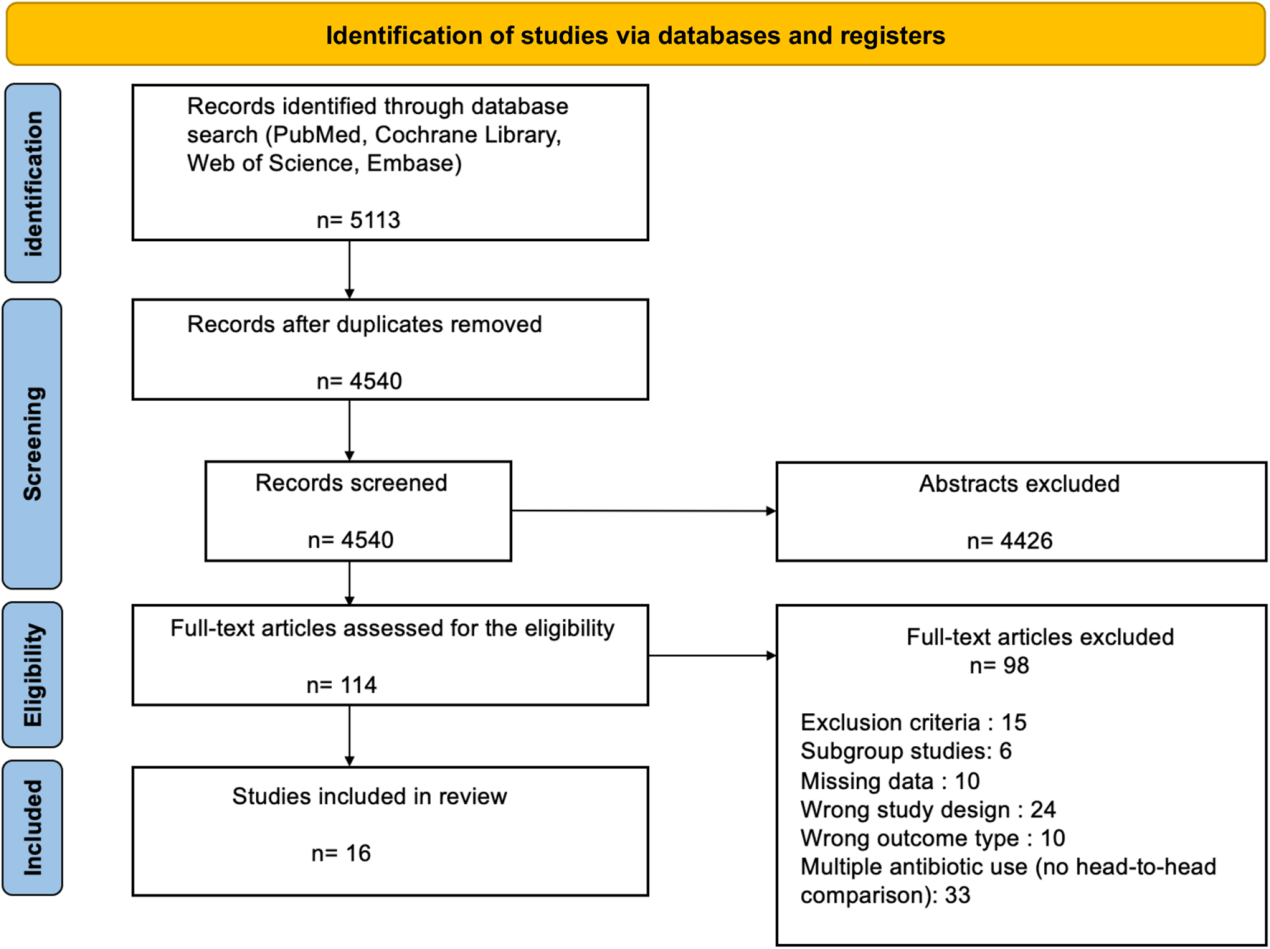
PRISMA diagram of the study selection process

A total of 5113 citations were initially identified, of which 4540 articles were screened. After screening for exclusion criteria, 114 studies were full text reviewed. Only RCTs met inclusion criteria, and all observational studies were excluded. The process of inclusion and exclusion is detailed in the PRISMA diagram (Fig. 1). Accordingly, 16 RCTs of 13 antibiotic regimens were included in the final analysis for the present NMA [9–12, 15–26].

References and characteristics of trials finally included are summarized in Table 1. A total of 4993 participants were included for the primary endpoint across 16 trials. Baseline characteristics were reported for 4568 patients of which 3215 (70.4%) were men, mean age was 59.5 years. VAP was found in 3121 (68.2%) patients enrolled in 15 of the 16 RCTs. Kidney failure, defined by a creatinine clearance < 60 ml/min, was reported in 451 patients. A bacterial pneumonia was documented in 2738 patients among 15 RCTs. Main pathogens identified were *K. pneumonia* (n = 745), *P. aeruginosa* (n = 651), *E. coli* (n = 361) and *A. baumannii* (n = 288). Clinical outcomes are compiled in Table 2.

**Table 2:** Clinical outcomes among the trials included

Author	Antibiotic regimen	Mortality rate, % (n/N)	Clinical cure, % (n/N)	Microbiological success, % (n/N)
Titov et al	Imipenem/Cilastatin/Relebactam	15.9 (42/264)	61 (161/264)	67.9 (146/215)
	Piperacillin/Tazobactam	21.4 (57/267)	55.8 (149/267)	61.9 (135/218)
Zanetti et al	Cefepime	25.9 (28/108)	70.4 (76/108)	61 (47/77)
	Imipenem/Cilastatin	18.8 (19/101)	74.3 (75/101)	53.5 (38/71)
Wunderink et al	Cefiderocol	20.7 (30/145)	64.8 (94/145)	40.7 (59/145)
	Meropenem	20.4 (30/147)	66.7 (98/147)	41.5 (61/147)
Torres et al	Ceftazidime/Avibactam	8.2 (29/356)	51.4 (132/257)	55.6 (95/171)
	Meropenem	6.8 (25/370)	48.5 (131/270)	64.1 (118/184)
Schmitt et al	Piperacillin/Tazobactam	15.9 (17/107)	59.8 (64/107)	34.6 (37/107)
	Imipenem/Cilastatin	10 (11/110)	66.4 (73/110)	46.4 (51/110)
Chastre et al	Doripenem	10.8 (27/249)	68.3 (86/126)	69 (80/116)
	Imipenem/Cilastatin	9.5 (24/252)	64.8 (79/122)	64.5 (71/110)
Kollef et al	Doripenem	20.9 (24/115)	45.6 (36/79)	45.6 (36/79)
	Imipenem/Cilastatin	14.3 (16/112)	56.8 (50/88)	56.8 (50/88)
Kollef et al	Ceftolozane/Tazobactam	24 (87/362)	54.4 (197/362)	53.3 (193/362)
	Meropenem	25.3 (92/364)	53.3 (194/364)	46.2 (168/364)
Cisneros et al	IV Colistin	22.5 (27/120)	68.3 (82/120)	56.1 (46/82)
	Meropenem	21.4 (24/112)	72.3 (81/112)	51.2 (42/82)
Réa-Neto et al	Doripenem	13.8 (30/217)	81.3 (109/134)	84.8 (84/99)
	Piperacillin/Tazobactam	14.6 (31/212)	79.8 (95/119)	80.6 (83/103)
Yamamato et al	Piperacillin/Tazobactam	2.9 (1/34)	75.9 (22/29)	84.2 (16/19)
	Meropenem	9.1 (3/33)	64.3 (18/28)	94.4 (17/18)
Lerma et al	Meropenem	23.2 (16/69)	56.5 (39/69)	55.1 (38/69)
	Ceftazidime	28.2 (20/71)	40.8 (29/71)	33.8 (24/71)
Torres et al	Ciprofloxacin	19.5 (8/41)	70.7 (29/41)	48.8 (20/41)
	Imipenem/Cilastatin	11.8 (4/34)	79.4 (27/34)	50 (17/34)
Abdelsalam et al	Colistin (IV)	43.3 (13/30)	56.7 (17/30)	*NA*
	Meropenem + Colistin (AS)	16.7 (5/30)	83.3 (25/30)	*NA*
Alvarez-Lerma et al	Piperacillin/Tazobactam	30.7 (27/88)	50 (44/88)	62 (31/50)
	Ceftazidime	22.2 (8/36)	44.4 (16/36)	65 (13/20)
Joshi et al	Piperacillin/Tazobactam	10.4 (23/222)	54.5 (121/222)	72.3 (112/155)
	Imipenem/Cilastatin	7.9 (17/215)	51.6 (111/215)	76.9 (93/121)

### Risk of bias assessment and certainty of evidence

**Fig. 2:**
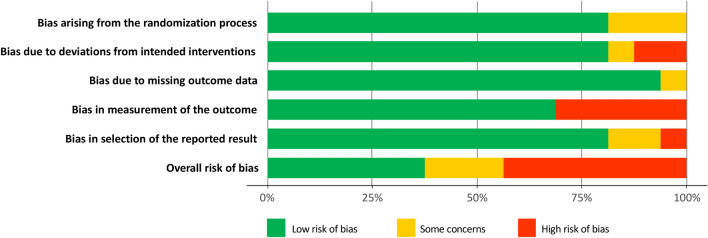
Summary of the risk of bias across all included trials

Thirteen trials provided a detailed and adequate randomization process. Two studies had a major deviation from the intended intervention [19, 26]. Only one trial suffered from concerns about missing data [16]. Nine trials were open-label studies and five were evaluated by investigator in charge of included patients, which were at high risk of bias in measurement of the outcome. The risk of bias was low, intermediate and high in six, three and seven trials respectively (Fig. 2). The certainty of evidence evaluating the head-to-head comparisons between different antibiotic regimens was assessed using the GRADE approach (Additional file 1: Table S1).

### Network

**Fig. 3:**
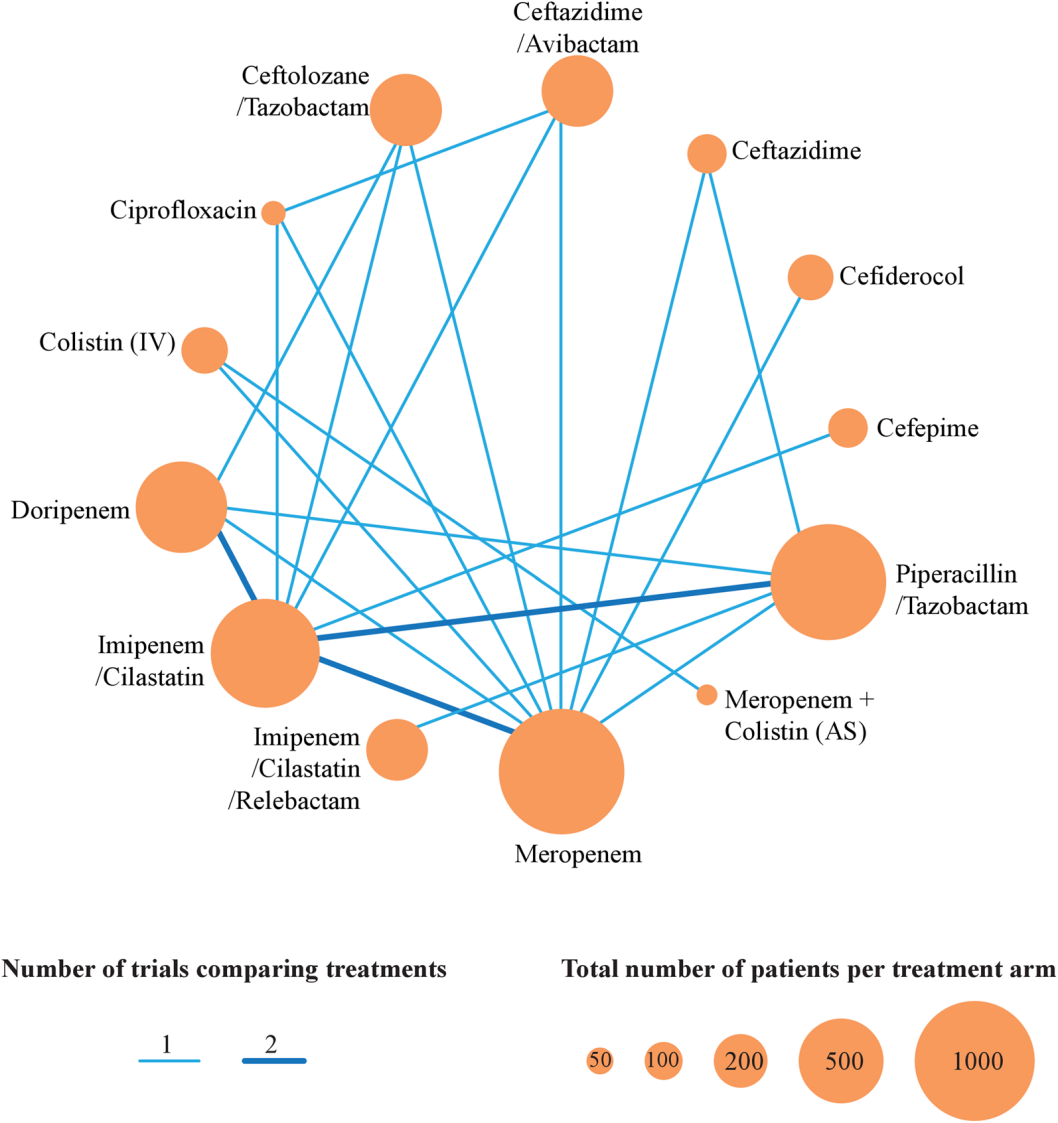
Network meta-analysis of eligible comparisons. Width and darkness of the lines are proportional to the number of trials comparing every pair of treatments and size of circles is proportional to the number of randomly assigned participants. *IV* intravenous, *AS* aerosolized

The visualization of network geometry of comparisons in all RCTs is shown in the Fig. 3. This network contained two closed loops consisting of three nodes and eight direct comparisons, which assessed a total of 13 antibiotic regimens.

### Primary outcome: 28-day mortality

**Fig. 4:**
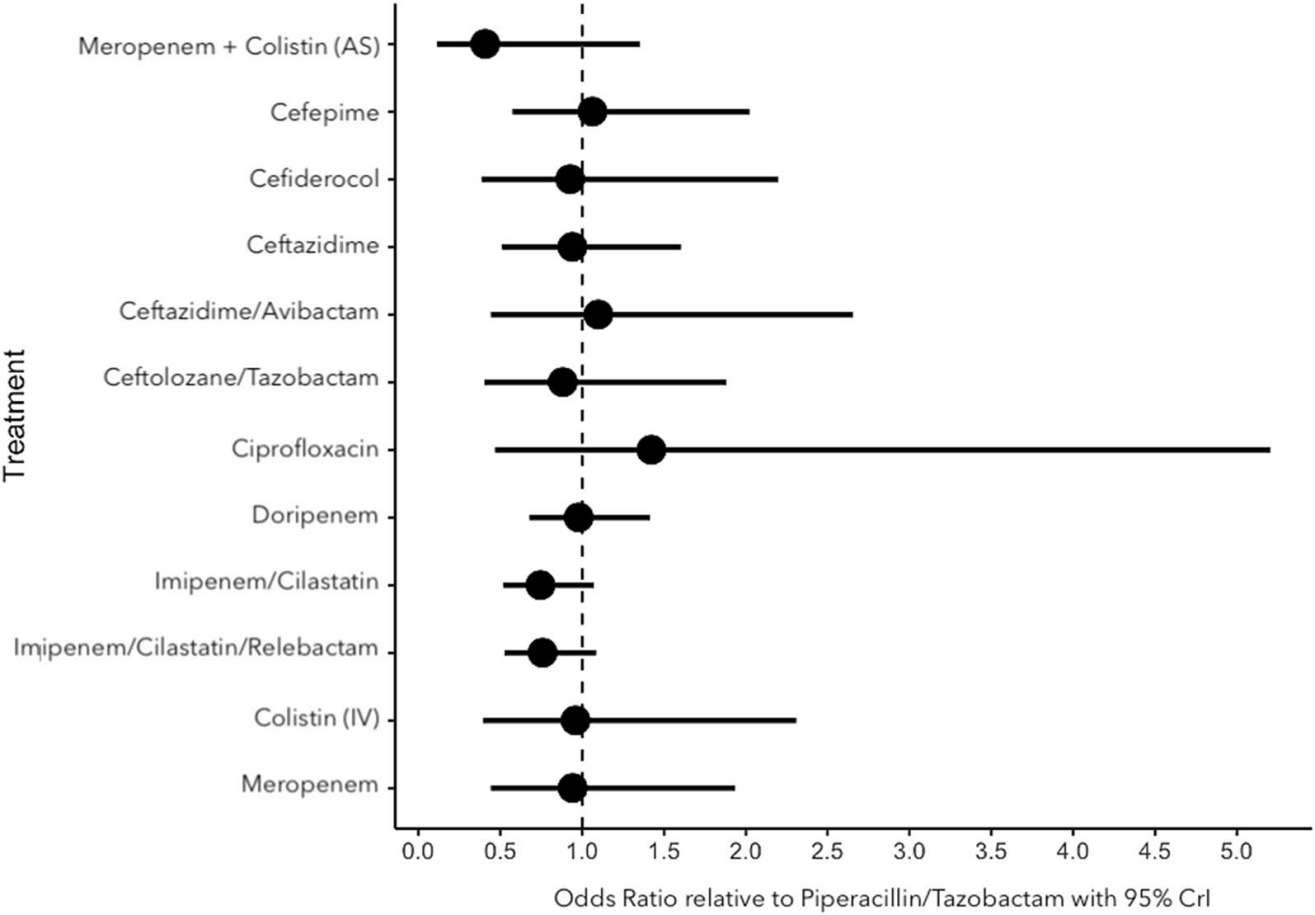
Forest plot for the primary outcome (28 day mortality) in all randomized controlled trials with odds-ratio (points) and their 95% CrIs (lines), using piperacillin/tazobactam as comparator

Twelve of the 16 RCTs reported precisely 28-day mortality as an endpoint. Two trials mentioned in-hospital mortality [16, 24] and two other trials reported crude mortality rates [23, 26]. Meropenem plus an adjunctive aerosolized colistin was associated with a significant decrease of mortality compared to colistin only (OR = 0.43; 95% CrI [0.17–0.94]). There were no significant differences for mortality among all others comparisons between antibiotic regimens. The results are summarized in Additional file 2: Fig. S1. None of the covariates improved the fit of the model in meta-regression analyses and therefore explained variation in treatment effects. Comparisons of antibiotic regimens using piperacillin/tazobactam as comparator are represented in Fig. 4.

The inconsistency of the model was tested on the direct comparison loops with a non-significant p-value (p = 0.96) allowing whole network estimates. The ranking of the probability for being the most effective treatment in terms of mortality is represented in Table 3. Meropenem plus aerosolized colistin had the highest likelihood of being top ranked (SUCRA = 92.0%), followed by imipenem/cilastatin (SUCRA = 72.2%) and imipenem/cilastatin/relebactam (SUCRA = 68.9%), while ciprofloxacin had the lowest (SUCRA = 24.0%). Assessment of transitivity is detailed in the Additional file 6. We found no concern about the transitivity across the comparisons. In addition, a subgroup analysis according to the risk of bias was not possible because no loop was present.

**Table 3:** SUCRA results in primary and secondary outcomes for the treatment of HCAP

Treatment	Mortality		Clinical success		Microbiological success		Adverse events	
	SUCRA	Rank	SUCRA	Rank	SUCRA	Rank	SUCRA	Rank
Ceftolozane/Tazobactam	55.2	4	43.6	9	89.6	1	61.5	6
Ceftazidime/Avibactam	33.1	12	51.5	7	35.9	10	71.2	2
Cefiderocol	48.6	5	32.6	11	59.2	5	66	3
Cefepime	36	11	52.6	6	69.1	3	17.1	11
Ceftazidime	47.5	6	4	13	9.9	12	64.2	5
Piperacillin/Tazobactam	38.8	10	58.6	5	19.3	11	35.9	10
Doripenem	41.9	9	64	4	37.7	9	53.4	7
Imipenem/Cilastatin	72.2	2	66	3	44.8	8	41	9
Imipenem/Cilastatin/Relebactam	68.9	3	80.7	2	45.8	6	45.1	8
Meropenem	46.9	7	38.7	10	66.7	4	74.5	1
Meropenem + Colistin (AS)	92	1	86.5	1	*NA*	*NA*	*NA*	*NA*
Colistin (IV)	45	8	26.9	12	76.7	2	65.2	4
Ciprofloxacin	24	13	44	8	45.3	7	4.8	12

### Secondary outcomes

#### Clinical cure

Data on clinical cure were available in all 16 included RCTs. Meropenem with (OR 1.97; 95% CrI [1.19–3.45]) or without aerosolized colistin (OR 1.40; 95% CrI [1.00–2.01]), imipemen/cilastatin/relebactam (OR 1.74; 95% CrI [1.03–2.90]) and ceftazidime/avibactam (OR 1.48; 95% CrI [1.02–2.20]) were associated with higher clinical cure rates compared to ceftazidime. Clinical cure was also higher in patients treated by meropenem plus aerosolized colistin in comparison with intravenous colistin alone (OR 1.48; 95% CrI [1.06–2.23]). There were no significant differences for clinical cure across the comparisons between other regimens (Additional file 3: Fig. S2). The inconsistency of the model was tested on the direct comparison loops with a non-significant p-value (p = 0.16) allowing whole network estimates. Meropenem plus aerosolized colistin (SUCRA 86.5%) and imipemen/cilastatin/relebactam (SUCRA 80.7%) were the two treatments with the highest probability of being superior to comparators, while ceftazidime had the lowest probability of being the most effective therapy (SUCRA 4.0%) (Table 3).

#### Microbiological cure

Microbiological cure was reported in 15 trials. In comparison with ceftazidime, ceftolozane/tazobactam (OR 1.62; 95% CrI [1.16–2.31]), intravenous colistin (OR 1.52; 95%CrI [1.01–2.35]) and meropenem (OR 1.41; 95% CrI [1.05–1.94]) were associated with increased microbiological cure. Patients treated by ceftolozane/tazobactam had higher microbiological cure than those treated by piperacillin/tazobactam (OR 1.42; 95% CrI [1.07–2.01]) or ceftazidime/avibactam (OR 1.33; 95% CrI [1.07–1.68]). All other comparisons between antibiotic regimens were not significant (Additional file 4: Fig. S3). The inconsistency of the model was tested on the direct comparison loops with a non-significant p-value (p = 0.06) allowing whole network estimates. Ceftolozane/tazobactam was the treatment with the highest probability of being superior to comparators (SUCRA 89.6%), while ceftazidime had the lowest (SUCRA 9.9%) (Table 3).

#### Adverse events

Adverse events were reported in 15 trials. There were no significant differences between treatments in the occurrence of at least one adverse event among trials. Meropenem had the highest probability of being the treatment with the fewest adverse events (SUCRA = 74.5%), while ciprofloxacin had the lowest probability (SUCRA = 4.8%) (Table 3). Information regarding the severe drug-related adverse events and drug-related discontinuation were reported in 10 and 8 trials, respectively. Additional analysis on these events were not allowed by NMA because the corresponding networks did not include any closed loop. Types of adverse events are specified in the Additional file 5: Table S2. Acute kidney injury (AKI) was reported in 13 of the 16 RCTs, and the greatest proportion of AKI occurred in patients treated by intravenous colistin (17%, N = 25/150). Data about epilepsy were available in 6 trials, of which imipenem/cilastatin had the most important seizure rate (4%, N = 10/263). Abnormal hepatic function and *Clostridium difficile* infections were reported in five and four studies respectively, and were uncommon (0.9, 0.8%).

## Discussion

This NMA including 16 trials evaluated the efficacy and the safety of 13 antibiotic regimens for the treatment of NP in approximately 5000 hospitalized patients. For 28 day mortality, we found no substantial differences between regimens. To our knowledge, this Bayesian NMA is the first to compare the efficacy of antibiotics targeting GNB in the setting of NP. In the field of nosocomial pneumonia, almost all RCTs are non-inferiority trials, using piperacillin or carbapenem as comparator. It is therefore unlikely that new antibiotics will be compared with each other in further large randomized trials. Based on direct and indirect comparisons, our work underlines important findings.

First, there were no significant differences between new antibiotics in terms of efficacy or safety. Importantly, the lack of difference among the antibiotic regimens does not imply that they are equal. In these non-inferiority trials, new antibiotics were started as empirical treatment of nosocomial pneumonia and pursued even if an antimicrobial de-escalation was possible. Current guidelines highly recommend the use of new BLBLI or cefiderocol in MDR/XDR infections after antibiotic stewardship considerations [27]. A major issue of this NMA is that analyses were unable to compare these new antibiotics in their real-life use in cases of bacterial resistance, considering intrinsic differences in spectrum or antimicrobial activity.

Interestingly, the only significant difference in mortality analysis was found in favor of using aerosolized colistin associated to meropenem over intravenous colistin. This result relies on a small trial which included 30 patients with VAP caused by MDR *K. pneumoniae* in each arm. This finding is consistent with previous trial, in which IV colistin alone showed a trend towards higher in-hospital mortality compared to a combination of IV/AS colistin in the treatment of MDRGNB in ICU [28]. Two observational studies and one NMA evaluating the adjunction of AS colistin found a benefit in clinical cure and microbiological eradication in the treatment of VAP [29–31]. The inferiority of IV colistin in the setting of pneumonia could be explained by its poor distribution into lung parenchyma (improved with inhaled use) and its nephrotoxicity [32]. Another hypothesis could be that this finding, derived from a small-sized RCT may indicate inconsistency within the network analysis. Indeed, most registration trials included hundreds of participants and targeted non-inferiority compared to a comparator, while this RCT (conducted in a MDR setting) is the only trial included in this NMA with significant differences in mortality rates between the two arms. This could lead to an overestimation of this result.

Microbiological data is a key issue and the source of heterogeneity among the included trials. Only one study was based on proven bacterial documentation at randomization [24]. To obtain culture-documentation is one of the challenges in nosocomial pneumonia, but the studies in this NMA reported high rates of isolated pathogens. For example, in RESTORE-IMI 2 and APEKS-NP trials, pneumonias were culture-documented in more than 80% of cases. However, real-life data showed that pathogens are identified on culture only in 50% of these patients [37]. This emphasizes the discrepancies between registration trials and clinical practice attributable to selection bias, introducing heterogeneity to the analyses.

This meta-analysis compiled data on various regimen of antibiotics targeting GNB. Across the last two decades, antibiotic’s resistance has raised dramatically, especially in *Enterobacterales*. For example, 3GCRE have emerged and represents one third of infections in some ICUs [33]. As a consequence, empirical treatment of NP relies on broad-spectrum antibiotics. This may explain why ceftazidime was associated with the lowest probability of clinical and microbiological cure despite its excellent activity against *P. aeruginosa*, including in the setting of respiratory infections [34]. Furthermore, it has been well-established that ceftazidime is inferior to comparators as empiric treatment in febrile neutropenic patients [35, 36]. On the other hand, our findings do not advocate for using the empirical antibiotic with the largest spectrum regimen available. Indeed, most regimens compared in this NMA had no significant differences between them in terms of mortality, clinical or microbiological cure.

The tolerability of antibiotics is another key element in the rationale of prescription. Because most antibiotics had similar clinical outcomes, the occurrence of adverse effects is even more important. Intravenous colistin is associated with important nephrotoxicity [37]. The two trials using intravenous colistin included on our NMA confirm this trend with 17% rates of acute renal failure [19, 24]. Another important issue is the neurologic tolerance to antibiotics, especially beta-lactams, colistin and quinolones. The occurrence of seizures were sparsely recorded in trials, but as expected, imipemen/cilastatin was the treatment with the highest risk of seizures (4%) [17]. Surprisingly, no epilepsy was reported among the 266 patients treated by imipenem/cilastatin/relebactam [11]. This could be related to the relatively low dose of imipenem (2 g/d) administered, which may limit the risk of seizures [38]. This suggests that antibiotics are well tolerated in the setting of nosocomial pneumonia, with the exception of intravenous colistin.

This study has limitations. First, among the 16 RCTs included in this NMA, only six (38%) were considered at low risk of bias. The network of these six trials did not contain a closed loop, which prevented us from performing sensitivity analyses to estimate difference of treatment effects in studies with low risk of bias. Second, this NMA did not integrate antibiotics dosages or treatment durations in the analyses. However, trials have demonstrated that a short treatment is non-inferior to longer antibiotic courses in nosocomial pneumonia [39–41]. Moreover, standard doses of antibiotics in lung infections are often sufficient, except for critically ills patients [42]. This is why these two variables not taken into account would probably not have had an impact on our analyses. Third, the network of this NMA contained more indirect than direct comparisons. In addition, heterogeneity among studies and the presence of outliers might have influenced the SUCRA analyses, potentially leading to instability in the findings related to efficacy and safety. Fourth, subgroup analyses according to the severity or to the bacteria would be clinically relevant but cannot be performed because of the lack of closed loop in dedicated networks. To address these points, an individual personalized data NMA should be performed. Fifth, registration trials in this meta-analysis excluded MDR/XDR pathogens. As a consequence, the findings of this meta-analysis may not be generalizable to difficult-to-treat resistant GNB infections in which antibiotic stewardship remains pivotal. All the limitations mentioned above lead to conditional findings.

## Conclusions

This NMA provides data suggesting that most of beta-lactams regimen had similar outcomes in terms of efficacy and safety, including new BLBLI, in the treatment of NP. Considering the very low to moderate certainty of evidence for the comparisons assessed in this meta-analysis, further studies are needed, especially in the field of multidrug-resistant Gram-negative bacterial pneumonia.

### Supplementary Information

**Additional file 1:** Appendix including PRISMA checklist.

**Additional file 2: Table S1.** Quality of evidence for primary endpoint using GRADEframework. **Table S2.** Summary of adverse events reported in trials included in the meta-analysis.

**Additional file 3: Figure S1.** Rank-heat plot of 28-day mortality of interventions in the treatment ofnosocomial pneumonia.

**Additional file 4: Figure S2.** Rank-heat plot of clinical cure of interventions in the treatment ofnosocomial pneumonia.

**Additional file 5: Figure S3.** Rank-heat plot of microbiological cure of interventions in the treatmentof nosocomial pneumonia.

**Additional file 6: Figure S4.** Rank-heat plot of adverse events of interventions in the treatment ofnosocomial pneumonia.

## Data Availability

All data in this study are available from the corresponding author (david.luque.paz@chu-rennes.fr) upon reasonable request.

## Abbreviations

MDR: Multidrug resistance
GNB: Gram-regative bacilli
3GCRE: Third-generation cephalosporin-resistant *Enterobacterales*
MDRGNB: Multidrug resistant Gram-negative bacteria
XDRGNB: Extensively drug resistant Gram-negative bacteria
NP: Nosocomial pneumonia
HAP: Hospitalized-acquired pneumonia
VAP: Ventilator-acquired pneumonia
BLBLI: Beta-lactam/beta-lactamase inhibitor
RCT: Randomized controlled trials
NMA: Network meta-analysis
PRISMA: Preferred Reporting Items for Systematic Reviews and Meta-Analyses
RoB: Risk of bias
OR: Odds Ratio
95% CrI: 95% Credible interval
SUCRA: Surface Under the Cumulative Ranking Curve
GRADE: Grading of Recommendations Assessment, Development and Evaluation
AKI: Acute kidney injury
