# Supplementary material for: Efficacy and safety of antibiotics targeting Gram-negative bacteria in nosocomial pneumonia: a systematic review and Bayesian network meta-analysis
Source: Ann Intensive Care. 2024 Apr 25;14:66. doi: 10.1186/s13613-024-01291-5 (PMC11045692; doi:10.1186/s13613-024-01291-5)

**Efficacy and safety of antibiotics targeting Gram negative bacteria in nosocomial pneumonia: a network meta-analysis**

Appendix

[Study screening and inclusion in the systematic review and meta-analysis 2](#_Toc128463580)

[Inclusion and exclusion criteria 3](#_Toc128463581)

[Reason for study exclusion through full-text screening 4](#_Toc128463582)

[PRISMA checklist for systematic review or meta-analysis 8](#_Toc128463583)

[Detailed risk of bias assessment 11](#_Toc128463584)

# Study screening and inclusion in the systematic review and meta-analysis

**Appendix Table 1**: Search strategy for study screening

| **Database** | **Search strategy** | **Accessed on (date)**  **Records identified** |
| --- | --- | --- |
| In PubMed database | ALL=((pneumonia) AND (adult) AND ((ventilator-associated) OR (nosocomial) OR (hospital-acquired) OR (health-care associated) OR (Gram-negative) OR (carbapenem resistant) OR (multidrug resistant)) AND ((amikacin) OR (tobra*) OR (genta*) OR (plazo*) OR (temocillin) OR (amoxicillin*) OR (clavulan*) OR (piperacillin*) OR (carbapenem) OR (meropenem) OR (ertapenem) OR (doripenem) OR (imipenem*) OR (biapenem) OR (panipenem) OR (cefuroxime) OR (ceftriaxone) OR (cefotaxime) OR (cefepime) OR (ceftaroline) OR (ceftolozane) OR (ceftazidime) OR (ceftobiprole) OR (cefiderocol) OR (aztreonam) OR (ciprofloxacin) OR (levofloxacin) OR (moxifloxacin) OR (tigecycline) OR (eravacycline) OR (colistin*) OR (avibactam) OR (vaborbactam) OR (relebactam) OR (tazobactam) OR (sulbactam)) | January 21, 2021  **2620 records** |
| In Cochrane database | ((pneumonia) AND (adult) AND ((ventilator-associated) OR (nosocomial) OR (hospital-acquired) OR (health-care associated) OR (Gram-negative) OR (carbapenem resistant) OR (multidrug resistant)) AND ((amikacin) OR (tobra*) OR (genta*) OR (plazo*) OR (temocillin) OR (amoxicillin*) OR (clavulan*) OR (piperacillin*) OR (carbapenem) OR (meropenem) OR (ertapenem) OR (doripenem) OR (imipenem*) OR (biapenem) OR (panipenem) OR (cefuroxime) OR (ceftriaxone) OR (cefotaxime) OR (cefepime) OR (ceftaroline) OR (ceftolozane) OR (ceftazidime) OR (ceftobiprole) OR (cefiderocol) OR (aztreonam) OR (ciprofloxacin) OR (levofloxacin) OR (moxifloxacin) OR (tigecycline) OR (eravacycline) OR (colistin*) OR (avibactam) OR (vaborbactam) OR (relebactam) OR (tazobactam) OR (sulbactam)) | January 21, 2021  **466 records** |
| In Web of Science database | (((pneumonia) AND (adult) AND ((ventilator-associated) OR (nosocomial) OR (hospital-acquired) OR (health care associated) OR (Gram-negative) OR (carbapenem resistant) OR (multidrug resistant)) AND ((amikacin) OR (tobra*) OR (genta*) OR (plazo*) OR (temocillin) OR (amoxicillin*) OR (clavulan*) OR (piperacillin*) OR (carbapenem) OR (meropenem) OR (ertapenem) OR (doripenem) OR (imipenem*) OR (biapenem) OR (panipenem) OR (cefuroxime) OR (ceftriaxone) OR (cefotaxime) OR (cefepime) OR (ceftaroline) OR (ceftolozane) OR(ceftazidime) OR (ceftobiprole) OR (cefiderocol) OR (aztreonam) OR (ciprofloxacin) OR (levofloxacin) OR (moxifloxacin) OR (tigecycline) OR (eravacycline) OR (colistin*) OR (avibactam) OR (vaborbactam) OR (relebactam) OR (tazobactam) OR (sulbactam)))) | January 21, 2021  **513 records** |
| In EMBASE database | ('pneumonia'/exp OR 'pneumonia') AND ('adult'/exp OR 'adult') AND ('ventilator-associated' OR 'nosocomial' OR 'hospital-acquired' OR 'health care associated' OR 'gram-negative' OR 'carbapenem resistant' OR 'multidrug resistant') AND ('amikacin'/exp OR 'amikacin' OR 'tobra*' OR 'genta*' OR 'plazo*' OR 'temocillin'/exp OR 'temocillin' OR 'amoxicillin*' OR 'clavulan*' OR 'piperacillin*' OR 'carbapenem'/exp OR 'carbapenem' OR 'meropenem'/exp OR 'meropenem' OR 'ertapenem'/exp OR 'ertapenem' OR 'doripenem'/exp OR 'doripenem' OR 'imipenem*' OR 'biapenem'/exp OR 'biapenem' OR 'panipenem'/exp OR 'panipenem' OR 'cefuroxime'/exp OR 'cefuroxime' OR 'ceftriaxone'/exp OR 'ceftriaxone' OR 'cefotaxime'/exp OR 'cefotaxime' OR 'cefepime'/exp OR 'cefepime' OR 'ceftaroline'/exp OR 'ceftaroline' OR 'ceftolozane'/exp OR 'ceftolozane' OR 'ceftazidime'/exp OR 'ceftazidime' OR 'ceftobiprole'/exp OR 'ceftobiprole' OR 'cefiderocol'/exp OR 'cefiderocol' OR 'aztreonam'/exp OR 'aztreonam' OR 'ciprofloxacin'/exp OR 'ciprofloxacin' OR 'levofloxacin'/exp OR 'levofloxacin' OR 'moxifloxacin'/exp OR 'moxifloxacin' OR 'tigecycline'/exp OR 'tigecycline' OR 'eravacycline'/exp OR 'eravacycline' OR 'colistin*' OR 'avibactam'/exp OR 'avibactam' OR 'vaborbactam'/exp OR 'vaborbactam' OR 'relebactam'/exp OR 'relebactam' OR 'tazobactam'/exp OR 'tazobactam' OR 'sulbactam'/exp OR 'sulbactam') | January 21, 2021  **1514 records** |

The search was restricted from January 2000 to December 2020. A standardized template was created in the Covidence platform, where references were imported for screening. After extraction of the four databases queries, 573 duplicates were automatically removed of the screening.

# Inclusion and exclusion criteria

Inclusion criteria were as follows:

- Randomized, or non-randomized, controlled or uncontrolled trials, and possibly observational studies
- Studies focusing on hospitalized adults
- Studies including patients with characterized healthcare associated pneumonia (HAP or VAP), suspected or confirmed to Gram-negative bacteria,
- Treated by empiric or adapted antibiotics
- Minimum sample size of studies: a minimum of 50 participants was required, 25 per each arm in a 1:1 trial or a total of 50 in the case of observational study.

Exclusion criteria were as follows:

- Studies including pediatric population (under 18 years of age)
- Studies assessing other infections than pneumonia
- Studies in which patients received multiple antibiotic regimens before or after inclusion (no head-to-head comparison)
- Studies with no report of mortality data
- Studies focusing on pneumonia due to Gram-positive bacteria
- Studies not written in the English language
- Studies conducted on animal models
- Subgroup analysis studies

# Reason for study exclusion through full-text screening

**Appendix Table 2: Details of studies excluded from full-text screening**

| **Title** | **Author** | **Reason for study exclusion** |
| --- | --- | --- |
| Management of ventilator associated pneumonia with a new antibiotic adjuvant entity (ceftriaxone+sulbactam+disodium edetate) - A novel approach to spare carbapenems. | Sathe P | Exclusion criteria |
| Meropenem/colistin versus meropenem/ampicillin-sulbactam in the treatment of carbapenem-resistant pneumonia. | Khalili H | Exclusion criteria |
| Safety and efficacy of colistin in Acinetobacter and Pseudomonas infections: a prospective cohort study. | Reina R; | Exclusion criteria |
| Carbapenem-resistant Pseudomonas aeruginosa pneumonia with intermediate minimum inhibitory concentrations to doripenem: combination therapy with high-dose, 4-h infusion of doripenem plus fosfomycin versus intravenous colistin plus fosfomycin. | Apisarnthanarak A | Exclusion criteria |
| Clinical activity of ceftazidime/avibactam against MDR Enterobacteriaceae and Pseudomonas aeruginosa: pooled data from the ceftazidime/avibactam Phase III clinical trial programme. | Stone GG | Exclusion criteria |
| Efficacy and safety of high-dose ampicillin/sulbactam vs. colistin as monotherapy for the treatment of multidrug resistant Acinetobacter baumannii ventilator-associated pneumonia | Betrosian | Exclusion criteria |
| Combination therapy versus monotherapy: a randomised pilot study on the evolution of inflammatory parameters after ventilator associated pneumonia [ISRCTN31976779]. | Damas P | Exclusion criteria |
| Once-daily cefepime versus ceftriaxone for nursing home-acquired pneumonia. | [Joseph A Paladino](https://pubmed.ncbi.nlm.nih.gov/?term=Paladino+JA&cauthor_id=17493183) | Exclusion criteria |
| Colistin versus colistin combined with ampicillin-sulbactam for multiresistant Acinetobacter baumannii ventilator-associated pneumonia treatment: An open-label prospective study | Makris, D | Exclusion criteria |
| Efficacy of cefepime versus ceftazidime in the treatment of adult pneumonia. | Lin JC | Exclusion criteria |
| Ceftaroline fosamil in the treatment of community-acquired bacterial pneumonia and acute bacterial skin and skin structure infections. | Lodise TP | Exclusion criteria |
| Interim Study: Comparison Of Safety And Efficacy of Levofloxacin Plus Colistin Regimen With Levofloxacin Plus High Dose Ampicillin/Sulbactam Infusion In Treatment of Ventilator-Associated Pneumonia Due To Multi Drug Resistant Acinetobacter | Mosaed R | Exclusion criteria |
| Phase III, randomized, multicentre, double-blind, double-dummy, parallel-group comparative study to determine the efficacy, safety and tolerability of ceftazidime-avibactam (CAZ-AVI) versus meropenem in the treatment of nosocomial pneumonia (N | Chow J | Exclusion criteria |
| (Efficacy and safety of levofloxacin infusion in patients with NHCAP categorized as group B or C according to the JRS Guidelines for the Management of Hospital-Acquired Pneumonia in Adults) | [Kei Yamasaki](https://pubmed.ncbi.nlm.nih.gov/?sort=date&term=Yamasaki+K&cauthor_id=24809206) | Exclusion criteria |
| Treatment of severe pneumonia in hospitalized patients: results of a multicenter, randomized, double-blind trial comparing intravenous ciprofloxacin with imipenem-cilastatin. The Severe Pneumonia Study Group | Fink, MP | Exclusion criteria |
| Comparison of the clinical efficacy between aerosolized colistin methanesulfonate plus tigecycline and aerosolized colistin methanesulfonate alone against extensively drug-resistant acinetobacter calcoaceticus-baumannii complex pneumonia | Hsieh, T.-C | Missing data |
| Carbapenem-resistant Acinetobacter baumannii pneumonia: Meropenem-rifampicin combination versus colistin | Song, J.Y | Missing data |
| Importance and efficacy of concurrent intravenous colistin with nebulized colistin for ventilator-associated pneumonia compared with only intravenous colistin | Kim, Y.J | Missing data |
| Comparison of intravenous colistin with and without aerosolized colistin for multi-drug resistant gram-negative pneumonia in critically ill patients | Doshi, N | Missing data |
| Treatment of ventilator-associated pneumonia with piperacillin-tazobactum and amikacin vs cefepime and levofloxacin: a randomized prospective study | Ahmed, S | Missing data |
| Clinical Benefits of Piperacillin/Tazobactam versus Ei a Combination of Ceftriaxone and Clindamycin in the Treatment of Early, Non-Ventilator, Hospital-Acquired Pneumonia in a Community-Based Hospital | Park GE | Missing data |
| Clinical efficacy of meropenem and imipenem in the treatment of elderly patients with nosocomial pneumonia | Jiang, L.-J | Missing data |
| Influence of amikacin inhalation on the efficacy of ventilation-associated pneumonia and ventilation-associated tracheobronchitis treatment caused by multi-drug resistant gram-negative bacteria: Comparative study | Yaroshetskiy, A. | Missing data |
| Temocillin (TMO) plus amoxicillin (AMX) for treatment of severe hospital acquired pneumonia (S-HAP) compared to piperacillin/tazobactam (PIP/TAZ) | Al-Dujaili, A | Missing data |
| Colistin-based versus sulbactam-based treatment for Acinetobacter baumannii bacteremic pneuomnia | Wang, Y.-C | Missing data |
| Randomized Trial of Ceftazidime-Avibactam vs Meropenem for Treatment of Hospital-Acquired and Ventilator-Associated Bacterial Pneumonia (REPROVE): Analyses per US FDA-Specified End Points. | Torres A | Subgroup studies |
| Medical resource utilization among patients with ventilator-associated pneumonia: pooled analysis of randomized studies of doripenem versus comparators. | Kollef MH | Subgroup studies |
| Ceftolozane/tazobactam (C/T) vs meropenem (MEM) in patients (pts) with ventilated hospital-acquired pneumonia (vHAP) - subset analysis of the ASPECT-NP randomized, controlled, phase 3 trial | Timsit, JF | Subgroup studies |
| Phase 3, Randomized, double-blind noninferiority (NI) study of ceftazidime-avibactam (CAZ-AVI) vs. meropenem (MER) in the treatment of patients with hospital-acquired bacterial pneumonia and ventilator-associated bacterial pneumonia (HABP/VABP): analyses | Torres, A | Subgroup studies |
| Outcomes in patients with ventilated nosocomial pneumonia (NP) and organ failure treated with ceftolozane/tazobactam (C/T) vs meropenem (MER) - analysis of the ASPECT-NP randomized, controlled trial | Martin-Loeches, I | Subgroup studies |
| An open-label, randomized and comparative study to evaluate the efficacy and safety of cefoperazone/sulbactam in comparison to cefepime for the treatment of hospital acquired pneumonia and healthcare-associated pneumonia | Wang, F-D | Subgroup studies |
| Efficacy and safety of cefiderocol or best available therapy for the treatment of serious infections caused by carbapenem-resistant Gram-negative bacteria (CREDIBLE-CR): a randomised, open-label, multicentre, pathogen-focused, descriptive, phase 3 trial. | Bassetti | Wrong comparator |
| Efficacy and toxicity of aerosolised colistin in ventilator-associated pneumonia: a prospective, randomised trial. | Abdellatif S | Wrong comparator |
| Active monotherapy and combination therapy for extensively drug-resistant Pseudomonas aeruginosa pneumonia. | Khawcharoenporn T | Wrong comparator |
| Clinical outcomes following treatment of Enterobacter species pneumonia with piperacillin/tazobactam compared to cefepime or ertapenem | Holsen MR | Wrong comparator |
| Is inhaled colistin beneficial in ventilator associated pneumonia or nosocomial pneumonia caused by Acinetobacter baumannii? | Demirdal T | Wrong comparator |
| Levofloxacin compared with imipenem/cilastatin followed by ciprofloxacin in adult patients with nosocomial pneumonia: a multicenter, prospective, randomized, open-label study. | West M | Wrong comparator |
| Comparison of ampicillin-sulbactam and imipenem-cilastatin for the treatment of acinetobacter ventilator-associated pneumonia | Wood GC | Wrong comparator |
| Addition of aerosolized colistin to the treatment of respiratory infection by multiresistant microorganisms | Maeso et al | Wrong comparator |
| Colistin monotherapy versus colistin-plus regimens for treatment of multi-drug resistant nosocomial pneumonia in critically ill patients | Eagleson N | Wrong comparator |
| Efficacy of nebulized colistin-based therapy without concurrent intravenous colistin for ventilator-associated pneumonia caused by carbapenem-resistant Acinetobacter baumannii | Kim, Y.K | Wrong comparator |
| Colistin plus carbapenem versus colistin monotherapy in the treatment of carbapenem-resistant acinetobacter baumannii pneumonia | Shi, H | Wrong comparator |
| Clinical experience of colistin-glycopeptide combination in critically ill patients infected with Gram-negative bacteria | Petrosillo N; | Wrong comparator |
| Randomized phase 2 trial to evaluate the clinical efficacy of two high-dosage tigecycline regimens versus imipenem-cilastatin for treatment of hospital-acquired pneumonia. | Ramirez J | Wrong comparator |
| Ertapenem versus cefepime for initial empirical treatment of pneumonia acquired in skilled-care facilities or in hospitals outside the intensive care unit | Yakovlev, S | Wrong comparator |
| Tigecycline-based versus sulbactam-based treatment for pneumonia involving multidrug-resistant Acinetobacter calcoaceticus-Acinetobacter baumannii complex. | Ye JJ | Wrong comparator |
| Outcomes in patients (pts) with failure of initial antibiotic therapy for hospital-acquired/ventilator-associated bacterial pneumonia (HABP/VABP) prior to enrollment in the phase 3 ASPECT-NP trial of ceftolozane/tazobactam (C/T) vs. meropenem (MEM) | Kollef, M | Wrong comparator |
| Colistin alone or combined with sulbactam or carbapenem against A. baumannii in ventilator-associated pneumonia. | Yilmaz GR | Wrong comparator |
| Comparable Efficacy of Tigecycline versus Colistin Therapy for Multidrug-Resistant and Extensively Drug-Resistant Acinetobacter baumannii Pneumonia in Critically Ill Patients. | Kim WY | Wrong comparator |
| Aerosolized Amikacin as Adjunctive Therapy of Ventilator-associated Pneumonia Caused by Multidrug-resistant Gram-negative Bacteria: A Single-center Randomized Controlled Trial. | Liu C | Wrong comparator |
| A randomized study of sequential intravenous/oral moxifloxacin in comparison to sequential intravenous ceftriaxone/oral cefuroxime axetil in patients with hospital-acquired pneumonia | Höffken G | Wrong comparator |
| Randomized controlled trial of nebulized colistimethate sodium as adjunctive therapy of ventilator-associated pneumonia caused by Gram-negative bacteria | Rattanaumpawan P | Wrong comparator |
| A Randomized Trial of the Amikacin Fosfomycin Inhalation System for the Adjunctive Therapy of Gram-Negative Ventilator-Associated Pneumonia: IASIS Trial. | Kollef MH | Wrong comparator |
| Efficacy and toxicity of high-dose nebulized colistin for critically ill surgical patients with ventilator-associated pneumonia caused by multidrug-resistant Acinetobacter baumannii. | Jang JY | Wrong comparator |
| Safety and efficacy of colistin compared with imipenem in the treatment of ventilator-associated pneumonia: a matched case-control study. | Kallel H | Wrong comparator |
| Colistin-based treatment for extensively drug-resistant Acinetobacter baumannii pneumonia | Khawcharoenporn T | Wrong comparator |
| Clinical outcomes of colistin in combination with either 6-G sulbactam or carbapenems for the treatment of extensively drug-resistant acinetobacter baumannii pneumonia with high MIC to sulbactam, a prospective cohort study | Ungthammakhun C | Wrong comparator |
| Inhaled colistin as adjunctive therapy to intravenous colistin for the treatment of microbiologically documented ventilator-associated pneumonia: a comparative cohort study. | Korbila IP | Wrong comparator |
| Treatment of Carbapenem-Resistant Acinetobacter baumannii Ventilator-Associated Pneumonia: Retrospective Comparison Between Intravenous Colistin and Intravenous Ampicillin-Sulbactam | Zalts R | Wrong comparator |
| Comparison of colistin and colistin/sulbactam for the treatment of multidrug resistant Acinetobacter baumannii ventilator-associated pneumonia | Kalin G | Wrong comparator |
| Inhaled amikacin adjunctive to intravenous standard-of-care antibiotics in mechanically ventilated patients with Gram-negative pneumonia (INHALE): a double-blind, randomised, placebo-controlled, phase 3, superiority trial. | Niederman MS | Wrong comparator |
| Randomized Phase 2 Trial To Evaluate the Clinical Efficacy of Two High-Dosage Tigecycline Regimens versus Imipenem-Cilastatin for Treatment of Hospital-Acquired Pneumonia | Julio Ramirez | Wrong comparator |
| Nebulized Versus IV Amikacin as Adjunctive Antibiotic for Hospital and Ventilator-Acquired Pneumonia Postcardiac Surgeries: A Randomized Controlled Trial. | Hassan NA | Wrong comparator |
| Clinical Benefits of Piperacillin/Tazobactam versus a Combination of Ceftriaxone and Clindamycin in the Treatment of Early, Non-Ventilator, Hospital-Acquired Pneumonia in a Community-Based Hospital. | Park GE; | Wrong comparator |
| Polymyxin for treatment of ventilator-associated pneumonia in a setting of high carbapenem resistance | Bento Talizin T | Wrong comparator |
| Randomized Noninferiority Trial of Cefoperazone-Sulbactam versus Cefepime in the Treatment of Hospital-Acquired and Healthcare-Associated Pneumonia. | Liu JW | Wrong outcome type |
| Efficacy and safety evaluation of fixed dose combination of cefepime and amikacin in comparison with cefepime alone in treatment of nosocomial pneumonia patients. | Chaudhary M | Wrong outcome type |
| Efficacy and safety of piperacillin/tazobactam versus biapenem in late elderly patients with nursing- and healthcare-associated pneumonia. | Karino F | Wrong outcome type |
| Levofloxacin for treatment of ventilator-associated pneumonia: a subgroup analysis from a randomized trial. | Shorr | Wrong outcome type |
| Treatment of Nosocomial Postoperative Pneumonia in Cancer Patients: A Prospective Randomized Study | Raad I | Wrong outcome type |
| Hospital-acquired pneumonia in general wards of a Japanese tertiary hospital | Ohi | Wrong outcome type |
| Reappraisal of clindamycin IV monotherapy for treatment of mild-to-moderate aspiration pneumonia in elderly patients. | Kadowaki M | Wrong outcome type |
| Effect of extended infusion of meropenem and nebulized amikacin on Gram-negative multidrug-resistant ventilator-associated pneumonia | Ammar, M | Wrong outcome type |
| Comparison of two therapeutic approaches for the management of ventilator-associated pneumonia due to multidrug-resistant Acinetobacter: a randomized clinical trial study | Khorvash F | Wrong outcome type |
| Imipenem, Meropenem, or Doripenem To Treat Patients with Pseudomonas aeruginosa Ventilator-Associated Pneumonia | Luyt CE | Wrong outcome type |
| A phase 3 randomized double-blind comparison of ceftobiprole medocaril versus ceftazidime plus linezolid for the treatment of hospital-acquired pneumonia. | Awad SS | Wrong study design |
| Ventilator-associated pneumonia in Iranian intensive care units. | Japoni A | Wrong study design |
| Comparison of the efficacy of colistin monotherapy and colistin combination therapies in the treatment of nosocomial pneumonia and ventilator-associated pneumonia caused by Acinetobacter baumannii | Kara, I | Wrong study design |
| Efficacy of high-dose nebulized colistin in ventilator-associated pneumonia caused by multidrug-resistant Pseudomonas aeruginosa and Acinetobacter baumannii. | Lu Q | Wrong study design |
| Prospective open-label randomized comparative, non-inferiority study of two initial antibiotic strategies for patients with nursing- and healthcare-associated pneumonia: Guideline-concordant therapy versus empiric therapy. | Matsuda S | Wrong study design |
| Randomized trial of combination versus monotherapy for the empiric treatment of suspected ventilator-associated pneumonia. | Heyland DK | Wrong study design |
| Comparison of the clinical efficacy between tigecycline plus extended-infusion imipenem and sulbactam plus imipenem against ventilator-associated pneumonia with pneumonic extensively drug-resistant Acinetobacter baumannii bacteremia, and correlation of clinical efficacy with in vitrosynergy tests | Jean SS | Wrong study design |
| Amoxicillin for acute lower respiratory tract infection in primary care: subgroup analysis of potential high-risk groups. | Moore | Wrong study design |
| Aerosolized colistin as adjunctive treatment of ventilator-associated pneumonia due to multidrug-resistant Gram-negative bacteria: a prospective study. | Micholopoulos | Wrong study design |
| Gentamicin therapy for sepsis due to carbapenem-resistant and colistin-resistant Klebsiella pneumoniae. | Gonzalez | Wrong study design |
| Acinetobacter baumannii ventilator-associated pneumonia: epidemiological and clinical findings. | Garnacho-Montero J; | Wrong study design |
| Effectiveness of tigecycline-based versus colistin- based therapy for treatment of pneumonia caused by multidrug-resistant Acinetobacter baumannii in a critical setting: a matched cohort analysis | Chuang YC | Wrong study design |
| Use of adjunctive aerosolized antimicrobial therapy in the treatment of Pseudomonas aeruginosa and Acinetobacter baumannii ventilator-associated pneumonia. | Arnold | Wrong study design |
| Colistin combination therapy improves microbiologic cure in critically ill patients with multi-drug resistant gram-negative pneumonia. | Parchem NL | Wrong study design |
| Pseudomonas aeruginosa ventilator-associated pneumonia. predictive factors of treatment failure. | Planquette B | Wrong study design |
| Effectiveness of azithromycin in aspiration pneumonia: a prospective observational study. | Marumo S | Wrong study design |
| Early use of imipenem/cilastatin and vancomycin followed by de-escalation versus conventional antimicrobials without de-escalation for patients with hospital-acquired pneumonia in a medical ICU: a randomized clinical trial. | Kim JW | Wrong study design |
| Is gentamicin safe and effective for severe community-acquired pneumonia? An 8-year retrospective cohort study. | Brereton CJ; | Wrong study design |
| Effect of aerosolized colistin as adjunctive treatment on the outcomes of microbiologically documented ventilator-associated pneumonia caused by colistin-only susceptible gram-negative bacteria. | Tumbarello M | Wrong study design |
| Comparative efficacy of doripenem versus meropenem for hospital-acquired and ventilator-associated pneumonia. | Liu WD | Wrong study design |
| Aerosolized plus intravenous colistin versus intravenous colistin alone for the treatment of ventilator-associated pneumonia: a matched case-control study | Kofteridis DP | Wrong study design |
| Amoxicillin plus temocillin as an alternative empiric therapy for the treatment of severe hospital-acquired pneumonia: results from a retrospective audit. | Habayeb H | Wrong study design |
| Hospital resource utilization with doripenem versus imipenem in the treatment of ventilator-associated pneumonia | Merchant S | Wrong study design |
| Comparison of tigecycline with imipenem/cilastatin for the treatment of hospital-acquired pneumonia | Freire AT | Wrong study design |

# PRISMA checklist for systematic review or meta-analysis

**Appendix Table 3**: The PRISMA checklist of items to report for systematic reviews incorporating network meta-analyses

| **Section/Topic** | **Item #** | **Checklist Item** | **Reported on Page #** |
| --- | --- | --- | --- |
| **TITLE** |  |  |  |
| Title | 1 | Identify the report as a systematic review incorporating a network meta-analysis*.* | 1 |
|  |  |  |  |
| **ABSTRACT** |  |  |  |
| Structured summary | 2 | Provide a structured summary including, as applicable:  **Background:** main objectives  **Methods:** data sources; study eligibility criteria, participants, and interventions; study appraisal; and synthesis methods, such as network meta-analysis.  **Results:** number of studies and participants identified; summary estimates with corresponding confidence/credible intervals*.*  **Discussion/Conclusions:** limitations; conclusions and implications of findings.  **Other:** primary source of funding; systematic review registration number with registry name. | 2 |
|  |  |  |  |
| **INTRODUCTION** |  |  |  |
| Rationale | 3 | Describe the rationale for the review in the context of what is already known, including mention of why a network meta-analysis has been conducted*.* | 3 |
| Objectives | 4 | Provide an explicit statement of questions being addressed, with reference to participants, interventions, comparisons, outcomes, and study design (PICOS). | 3,5 |
|  |  |  |  |
| **METHODS** |  |  |  |
| Protocol and registration | 5 | Indicate whether a review protocol exists and if and where it can be accessed (e.g., Web address); and, if available, provide registration information, including registration number. | 4 |
| Eligibility criteria | 6 | Specify study characteristics (e.g., PICOS, length of follow-up) and report characteristics (e.g., years considered, language, publication status) used as criteria for eligibility, giving rationale. Clearly describe eligible treatments included in the treatment network, and note whether any have been clustered or merged into the same node (with justification). | 4,5 |
| Information sources | 7 | Describe all information sources (e.g., databases with dates of coverage, contact with study authors to identify additional studies) in the search and date last searched. | 4,5 & SM |
| Search | 8 | Present full electronic search strategy for at least one database, including any limits used, such that it could be repeated. | 4,5 & SM |
| Study selection | 9 | State the process for selecting studies (i.e., screening, eligibility, included in systematic review, and, if applicable, included in the meta-analysis). | 4,5 |
| Data collection process | 10 | Describe method of data extraction from reports (e.g., piloted forms, independently, in duplicate) and any processes for obtaining and confirming data from investigators. | 5 |
| Data items | 11 | List and define all variables for which data were sought (e.g., PICOS, funding sources) and any assumptions and simplifications made. | 5 |
| **Geometry of the network** | **S1** | Describe methods used to explore the geometry of the treatment network under study and potential biases related to it. This should include how the evidence base has been graphically summarized for presentation, and what characteristics were compiled and used to describe the evidence base to readers. | 5-7 & SM |
| Risk of bias within individual studies | 12 | Describe methods used for assessing risk of bias of individual studies (including specification of whether this was done at the study or outcome level), and how this information is to be used in any data synthesis. | 5 |
| Summary measures | 13 | State the principal summary measures (e.g., risk ratio, difference in means). Also describe the use of additional summary measures assessed, such as treatment rankings and surface under the cumulative ranking curve (SUCRA) values, as well as modified approaches used to present summary findings from meta-analyses*.* | 6-7 &SM |
| Planned methods of analysis | 14 | Describe the methods of handling data and combining results of studies for each network meta-analysis. This should include, but not be limited to:   - Handling of multi-arm trials; - Selection of variance structure; - Selection of prior distributions in Bayesian analyses; and - Assessment of model fit*.* | 6-7 |
| **Assessment of Inconsistency** | **S2** | Describe the statistical methods used to evaluate the agreement of direct and indirect evidence in the treatment network(s) studied. Describe efforts taken to address its presence when found. | 6 |
| Risk of bias across studies | 15 | Specify any assessment of risk of bias that may affect the cumulative evidence (e.g., publication bias, selective reporting within studies). | 5,6 & SM |
| Additional analyses | 16 | Describe methods of additional analyses if done, indicating which were pre-specified. This may include, but not be limited to, the following:   - Sensitivity or subgroup analyses; - Meta-regression analyses; - Alternative formulations of the treatment network; and - Use of alternative prior distributions for Bayesian analyses (if applicable). | 6 |
|  |  |  |  |
| **RESULTS†** |  |  |  |
| Study selection | 17 | Give numbers of studies screened, assessed for eligibility, and included in the review, with reasons for exclusions at each stage, ideally with a flow diagram. | 7 |
| **Presentation of network structure** | **S3** | Provide a network graph of the included studies to enable visualization of the geometry of the treatment network. | Fig. 1 |
| **Summary of network geometry** | **S4** | Provide a brief overview of characteristics of the treatment network. This may include commentary on the abundance of trials and randomized patients for the different interventions and pairwise comparisons in the network, gaps of evidence in the treatment network, and potential biases reflected by the network structure. | Table 1 |
| Study characteristics | 18 | For each study, present characteristics for which data were extracted (e.g., study size, PICOS, follow-up period) and provide the citations. | Table 1 |
| Risk of bias within studies | 19 | Present data on risk of bias of each study and, if available, any outcome level assessment. | Fig. 3 |
| Results of individual studies | 20 | For all outcomes considered (benefits or harms), present, for each study: 1) simple summary data for each intervention group, and 2) effect estimates and confidence intervals. Modified approaches may be needed to deal with information from larger networks. | Table 1 & SM |
| Synthesis of results | 21 | Present results of each meta-analysis done, including confidence/credible intervals. In larger networks, authors may focus on comparisons versus a particular comparator (e.g. placebo or standard care), with full findings presented in an appendix. League tables and forest plots may be considered to summarize pairwise comparisons*.* If additional summary measures were explored (such as treatment rankings), these should also be presented. | 7-9 |
| **Exploration for inconsistency** | **S5** | Describe results from investigations of inconsistency. This may include such information as measures of model fit to compare consistency and inconsistency models, *P* values from statistical tests, or summary of inconsistency estimates from different parts of the treatment network. | 7-9 |
| Risk of bias across studies | 22 | Present results of any assessment of risk of bias across studies for the evidence base being studied. | 7 & SM |
| Results of additional analyses | 23 | Give results of additional analyses, if done (e.g., sensitivity or subgroup analyses, meta-regression analyses*,* alternative network geometries studied, alternative choice of prior distributions for Bayesian analyses, and so forth). | NA |
|  |  |  |  |
| **DISCUSSION** |  |  |  |
| Summary of evidence | 24 | Summarize the main findings, including the strength of evidence for each main outcome; consider their relevance to key groups (e.g., healthcare providers, users, and policy-makers). | 10 |
| Limitations | 25 | Discuss limitations at study and outcome level (e.g., risk of bias), and at review level (e.g., incomplete retrieval of identified research, reporting bias). Comment on the validity of the assumptions, such as transitivity and consistency. Comment on any concerns regarding network geometry (e.g., avoidance of certain comparisons*).* | 13 |
| Conclusions | 26 | Provide a general interpretation of the results in the context of other evidence, and implications for future research. | 13 |
|  |  |  |  |
| **FUNDING** |  |  |  |
| Funding | 27 | Describe sources of funding for the systematic review and other support (e.g., supply of data); role of funders for the systematic review. This should also include information regarding whether funding has been received from manufacturers of treatments in the network and/or whether some of the authors are content experts with professional conflicts of interest that could affect use of treatments in the network. | 14 |

PICOS = population, intervention, comparators, outcomes, study design.

† Authors may wish to plan for use of appendices to present all relevant information in full detail for items in this section.

# Detailed risk of bias assessment

Details of the risk of bias assessed using ‘RoB 2’: a revised Cochrane risk-of-bias tool for randomized trials.


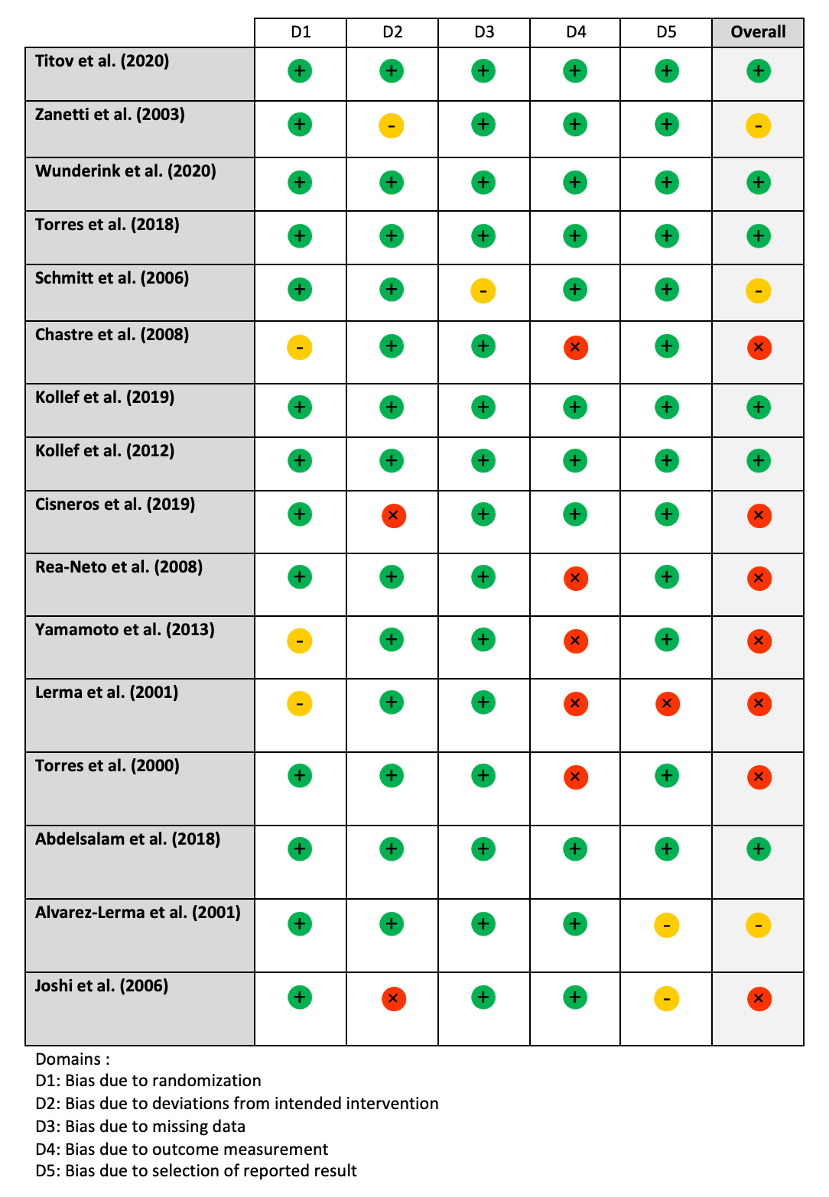

Supplement: Supplementary file 1 — Additional file 1: Appendix including PRISMA checklist. [file 13613_2024_1291_MOESM1_ESM.docx]
