# Supplementary material for: Efficacy and safety of antibiotics targeting Gram-negative bacteria in nosocomial pneumonia: a systematic review and Bayesian network meta-analysis
Source: Ann Intensive Care. 2024 Apr 25;14:66. doi: 10.1186/s13613-024-01291-5 (PMC11045692; doi:10.1186/s13613-024-01291-5)
Supplement: Supplementary file 2 — Additional file 2: Table S1. Quality of evidence for primary endpoint using GRADEframework. Table S2. Summary of adverse events reported in trials included in the meta-analysis. [file 13613_2024_1291_MOESM2_ESM.docx]

**Table S1**. Grades of Recommendations, Assessment, Development and Evaluations (GRADE) using day-28 mortality as primary outcome.

| Comparison | Certainty assessment | | | | | | | Odds-ratio  (95% CI) | Certainty |
| --- | --- | --- | --- | --- | --- | --- | --- | --- | --- |
|  | Number of studies | Study design | Risk of bias | Inconsistency | Indirectness | Imprecision | Publication bias |  |  |
| Imipenem/Cilastatin/Relebactam *vs* Piperacillin-Tazobactam | 1 | RCT | Not Serious | Serious ^a^ | Not Serious | Not Serious | No downgrade | 1.32 (0.92 -1.90) | MODERATE |
| Cefepime *vs* Imipenem/Cilastatin | 1 | RCT | Serious | Serious ^a^ | Serious | Serious | No downgrade | 0.70 (0.41- 1.16) | VERY LOW |
| Cefiderocol *vs* Meropenem | 1 | RCT | Not Serious | Serious ^a^ | Not Serious | Not Serious | No downgrade | 1.01 (0.64 - 1.58) | MODERATE |
| Ceftazidime/Avibactam *vs* Meropenem | 1 | RCT | Not Serious | Serious ^a^ | Serious | Not Serious | No downgrade | 0.86 (0.51 - 1.44) | LOW |
| Piperacillin/Tazobactam *vs* Imipenem/Cilastatin | 2 | RCT | Serious | Not Serious | Serious | Serious | No downgrade | 0.75 (0.52 - 1.07) | LOW |
| Doripenem *vs* Imipenem/Cilastatin | 2 | RCT | Not Serious | Not Serious | Serious | Not Serious | No downgrade | 0.76 (0.55 - 1.05) | MODERATE |
| Ceftolozane/Tazobactam *vs* Meropenem | 1 | RCT | Not Serious | Serious ^a^ | Not Serious | Not Serious | No downgrade | 1.06 (0.83 - 1.38) | MODERATE |
| Colistin (intravenous) *vs* Meropenem | 1 | RCT | Serious | Serious ^a^ | Not Serious | Serious | No downgrade | 0.98 (0.60 - 1.58) | VERY LOW |
| Colistin (intravenous) vs Meropenem plus Colistin (aerosolized) | 1 | RCT | Serious | Serious ^a^ | Not Serious | Serious | No downgrade | 0.43 (0.17 - 0.94) | VERY LOW |
| Doripenem *vs* Piperacillin/Tazobactam | 1 | RCT | Serious | Serious ^a^ | Serious | Not Serious | No downgrade | 1.02 (0.71 - 1.47) | VERY LOW |
| Meropenem *vs* Piperacillin/Tazobactam | 1 | RCT | Serious | Serious ^a^ | Serious | Serious | No downgrade | 1.06 (0.52 - 2.26) | VERY LOW |
| Ceftazidime *vs* Piperacillin/Tazobactam | 1 | RCT | Not Serious | Serious ^a^ | Serious | Serious | No downgrade | 1.06 (0.62 - 1.96) | VERY LOW |
| Meropenem *vs* Ceftazidime | 1 | RCT | Serious | Serious ^a^ | Serious | Serious | No downgrade | 1.00 (0.58 - 1.71) | VERY LOW |
| Imipenem/Cilastatin vs Ciprofloxacin | 1 | RCT | Serious | Serious ^a^ | Serious | Serious | No downgrade | 1.90 (0.66 - 6.71) | VERY LOW |

a : Downgraded by one when unable to evaluate inconsistency/heterogeneity due to lack of sufficient data (a single study)

**Tables S2**. Summary of adverse events reported in trials included in the meta-analysis

| Study | Antibiotic regimen | Nb of patients (safety population) | Patients w/ at least one adverse effect | Severe AE | Drug related AE | Severe drug-related AE | Discon-tinuation due to AE | Drug-related discontinuation after AE | Thombo-  cytopenia | Diarrhea | Vomiting | Anormal hepatic function | Cholesteasis | Elevated liver enzymes | Skin disorders | Seizures | *C. difficile* infection | AKI injury |
| --- | --- | --- | --- | --- | --- | --- | --- | --- | --- | --- | --- | --- | --- | --- | --- | --- | --- | --- |
| Titov *et al*. | Imipenem/Cilastatin/Relebactam | 266 | 226 | 71 | 31 | 3 | 15 | 6 | 1 | 6 | 2 | 1 | *NA* | 6 | 6 | 0 | 1 | 0 |
|  | Piperacillin/Tazobactam | 269 | 233 | 86 | 26 | 2 | 22 | 4 | 0 | 6 | 1 | 0 | *NA* | 3 | 8 | 1 | 0 | 1 |
| Zanetti *et al*. | Cefepime | 138 | 71 | 14 | 33 | 1 | *NA* | *NA* | *NA* | 7 | *NA* | *NA* | *NA* | *NA* | *NA* | *NA* | 0 | 5 |
|  | Imipenem/Cilastatin | 141 | 62 | 8 | 20 | 0 | *NA* | *NA* | *NA* | 9 | *NA* | *NA* | *NA* | *NA* | *NA* | *NA* | 6 | 0 |
| Wunderink *et al.* | Cefiderocol | 148 | 130 | 56 | 14 | 3 | 12 | 2 | 0 | 3 | *NA* | 1 | *NA* | 2 | 1 | *NA* | 1 | 0 |
|  | Meropenem | 150 | 129 | 45 | 17 | 5 | 14 | 1 | 1 | 5 | *NA* | 1 | *NA* | 1 | 0 | *NA* | 2 | 0 |
| Torres *et al.* | Ceftazidime/Avibactam | 405 | 302 | 75 | *NA* | 4 | 16 | 2 | *NA* | *NA* | 1 | *NA* | 1 | 2 | 0 | 1 | *NA* | 2 |
|  | Meropenem | 403 | 299 | 54 | *NA* | 0 | 11 | 0 | *NA* | *NA* | 0 | *NA* | 0 | 1 | 3 | 0 | *NA* | 2 |
| Schmitt *et al.* | Piperacillin/Tazobactam | 110 | 82 | 25 | 33 | 4 | 27 | 13 | *NA* | 11 | 0 | *NA* | 0 | *NA* | *NA* | *NA* | *NA* | 4 |
|  | Imipenem/Cilastatin | 111 | 72 | 21 | 28 | 4 | 15 | 9 | *NA* | 3 | 4 | *NA* | 5 | *NA* | *NA* | *NA* | *NA* | 0 |
| Chastre *et al.* | Doripenem | 262 | 72 | *NA* | 45 | 5 | 17 | *NA* | *NA* | 5 | 4 | 2 | *NA* | 12 | 5 | 3 | *NA* | *NA* |
|  | Imipenem/Cilastatin | 263 | 72 | *NA* | 46 | 4 | 15 | *NA* | *NA* | 8 | 2 | 4 | *NA* | 6 | 2 | 10 | *NA* | *NA* |
| Kollef *et al.* | Doripenem | 115 | 106 | *NA* | *NA* | *NA* | *NA* | *NA* | 4 | 11 | 6 | *NA* | 4 | *NA* | *NA* | *NA* | *NA* | 3 |
|  | Imipenem/Cilastatin | 112 | 107 | *NA* | *NA* | *NA* | *NA* | *NA* | 5 | 13 | 6 | *NA* | 2 | *NA* | *NA* | *NA* | *NA* | 6 |
| Kollef *et al.* | Ceftolozane/Tazobactam | 361 | 310 | 143 | 38 | 8 | 37 | 4 | *NA* | 4 | 2 | 1 | 0 | 12 | 2 | *NA* | 4 | 1 |
|  | Meropenem | 359 | 299 | 136 | 27 | 2 | 42 | 5 | *NA* | 6 | 1 | 0 | 1 | 5 | 2 | *NA* | 1 | 0 |
| Cisneros *et al.* | IV Colistin | 120 | 50 | 11 | *NA* | 4 | *NA* | *NA* | *NA* | *NA* | *NA* | *NA* | *NA* | *NA* | *NA* | *NA* | *NA* | 20 |
|  | Meropenem | 112 | 45 | 2 | *NA* | 1 | *NA* | *NA* | *NA* | *NA* | *NA* | *NA* | *NA* | *NA* | *NA* | *NA* | *NA* | 9 |
| Réa-Neto *et al.* | Doripenem | 223 | 36 | 67 | 36 | 0 | 9 | *NA* | 4 | 4 | *NA* | *NA* | *NA* | 4 | *NA* | 3 | *NA* | *NA* |
|  | Piperacillin/Tazobactam | 221 | 39 | 58 | 39 | 0 | 14 | *NA* | 5 | 5 | *NA* | *NA* | *NA* | 2 | *NA* | 6 | *NA* | *NA* |
| Yamamato *et al.* | Piperacillin/Tazobactam | 49 | 11 | 0 | *NA* | 0 | 0 | 0 | *NA* | 3 | *NA* | 3 | *NA* | *NA* | *NA* | *NA* | *NA* | 0 |
|  | Meropenem | 46 | 8 | 0 | *NA* | 0 | 0 | 0 | *NA* | 1 | *NA* | 7 | *NA* | *NA* | *NA* | *NA* | *NA* | 0 |
| Lerma *et al.* | Meropenem | 69 | 31 | 23 | 7 | *NA* | *NA* | 3 | *NA* | 1 | *NA* | *NA* | 1 | 2 | 2 | 1 | *NA* | 0 |
|  | Ceftazidime | 71 | 35 | 32 | 12 | *NA* | *NA* | 4 | *NA* | 1 | *NA* | *NA* | 0 | 5 | 1 | 0 | *NA* | 2 |
| Torres *et al.* | Ciprofloxacin | 72 | 21 | *NA* | 6 | *NA* | *NA* | *NA* | 0 | 0 | *NA* | *NA* | *NA* | *NA* | 0 | 0 | *NA* | 1 |
|  | Imipenem/Cilastatin | 77 | 14 | *NA* | 4 | *NA* | *NA* | *NA* | 0 | 0 | *NA* | *NA* | *NA* | *NA* | 0 | 0 | *NA* | 0 |
| Abdelsalam *et al.* | IV Colistin | 30 | *NA* | *NA* | *NA* | *NA* | *NA* | *NA* | 4 | *NA* | *NA* | *NA* | *NA* | 3 | *NA* | *NA* | *NA* | 5 |
|  | Meropenem + AS Colistin | 30 | *NA* | *NA* | *NA* | *NA* | *NA* | *NA* | 6 | *NA* | *NA* | *NA* | *NA* | 4 | *NA* | *NA* | *NA* | 4 |
| Alvarez-Lerma *et al.* | Piperacillin/Tazobactam | 88 | 21 | *NA* | *NA* | *NA* | *NA* | *NA* | *NA* | 3 | 0 | *NA* | *NA* | 3 | 1 | *NA* | *NA* | 6 |
|  | Ceftazidime | 36 | 5 | *NA* | *NA* | *NA* | *NA* | *NA* | *NA* | 0 | 1 | *NA* | *NA* | 0 | 0 | *NA* | *NA* | 2 |
| Joshi *et al.* | Piperacillin/Tazobactam | 222 | 204 | 42 | *NA* | *NA* | 25 | 0 | *NA* | *NA* | *NA* | *NA* | *NA* | *NA* | *NA* | *NA* | *NA* | *NA* |
|  | Imipenem/Cilastatin | 215 | 198 | 41 | *NA* | *NA* | 14 | 2 | *NA* | *NA* | *NA* | *NA* | *NA* | *NA* | *NA* | *NA* | *NA* | *NA* |
