# Supplementary figures and images for: Efficacy and safety of antibiotics targeting Gram-negative bacteria in nosocomial pneumonia: a systematic review and Bayesian network meta-analysis
Source: Ann Intensive Care. 2024 Apr 25;14:66. doi: 10.1186/s13613-024-01291-5 (PMC11045692; doi:10.1186/s13613-024-01291-5)

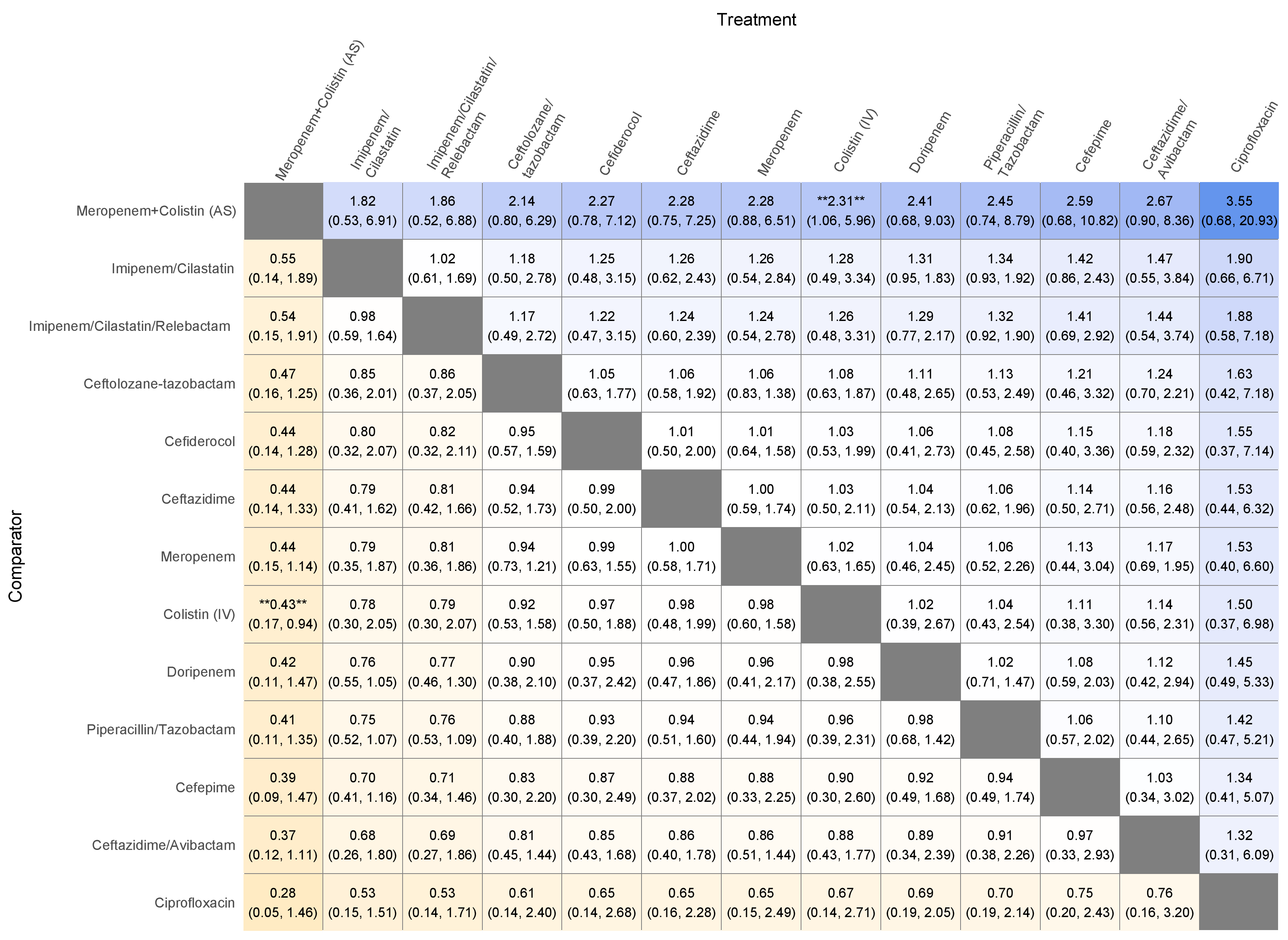

Supplement: Supplementary file 3 — Additional file 3: Figure S1. Rank-heat plot of 28-day mortality of interventions in the treatment ofnosocomial pneumonia. [file 13613_2024_1291_MOESM3_ESM.tiff]

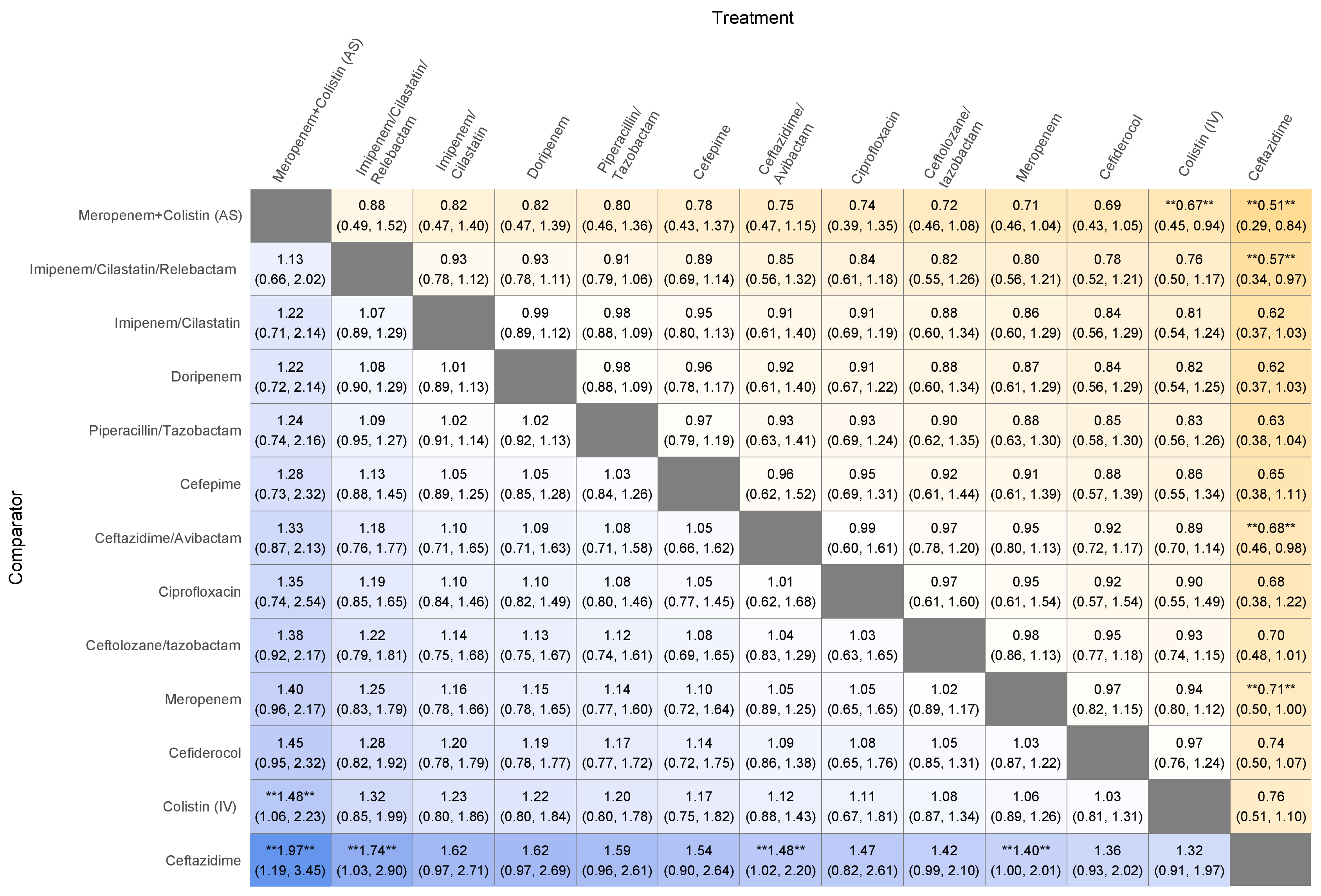

Supplement: Supplementary file 4 — Additional file 4: Figure S2. Rank-heat plot of clinical cure of interventions in the treatment ofnosocomial pneumonia. [file 13613_2024_1291_MOESM4_ESM.tiff]

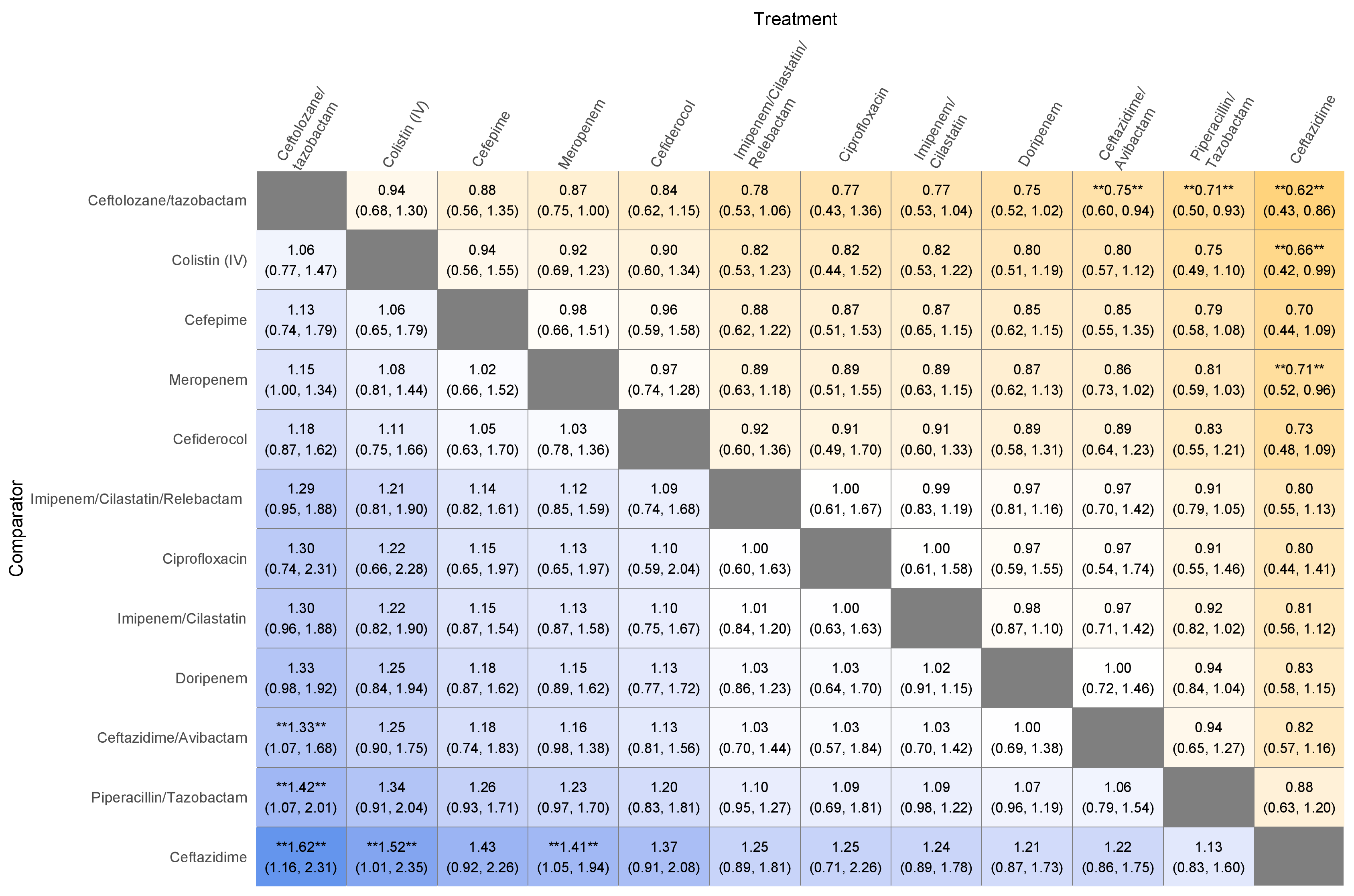

Supplement: Supplementary file 5 — Additional file 5: Figure S3. Rank-heat plot of microbiological cure of interventions in the treatmentof nosocomial pneumonia. [file 13613_2024_1291_MOESM5_ESM.tiff]

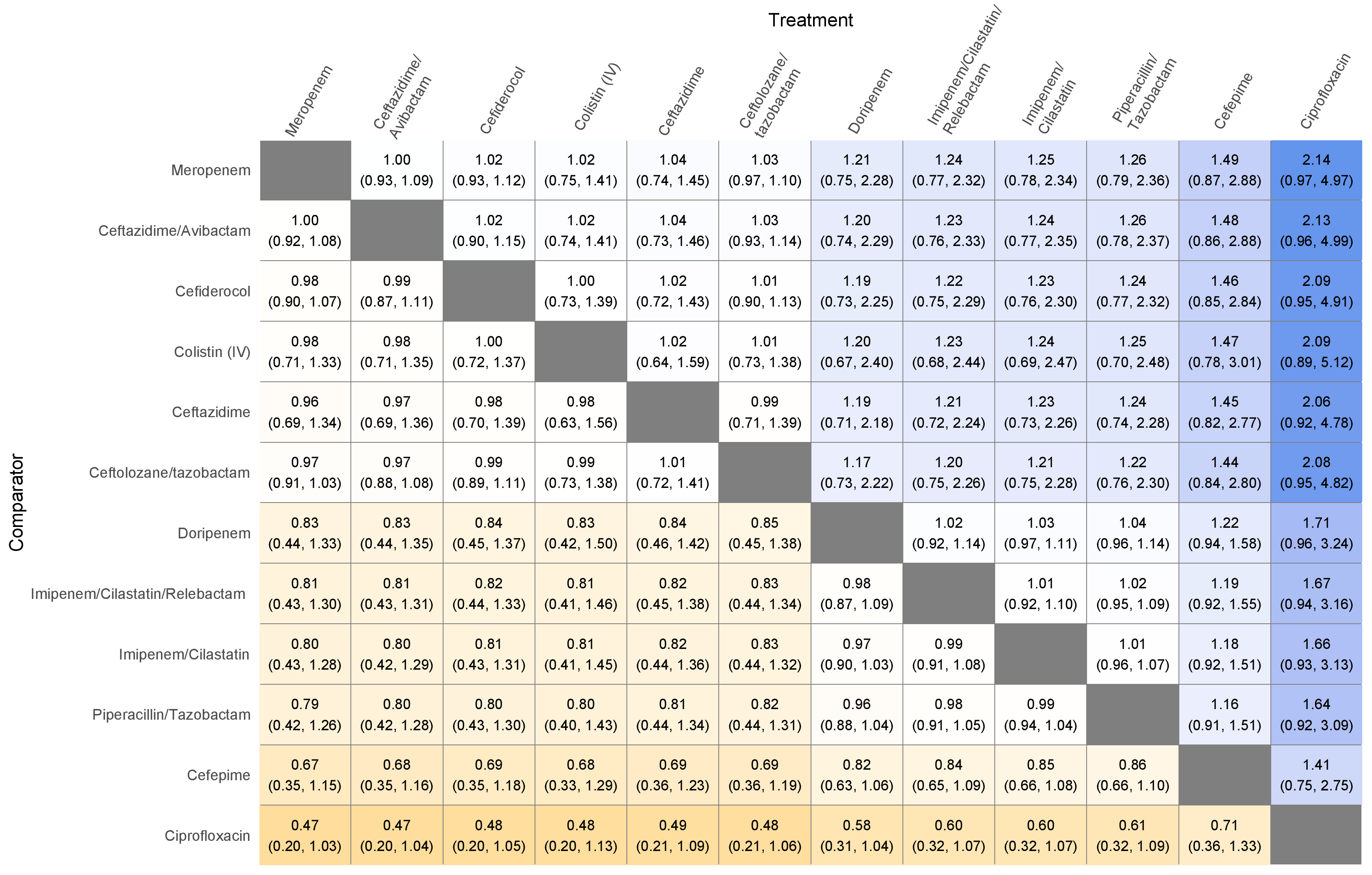

Supplement: Supplementary file 6 — Additional file 6: Figure S4. Rank-heat plot of adverse events of interventions in the treatment ofnosocomial pneumonia. [file 13613_2024_1291_MOESM6_ESM.tiff]
